# The nucleosome acidic patch and H2A ubiquitination underlie mSWI/SNF recruitment in synovial sarcoma

**DOI:** 10.1038/s41594-020-0466-9

**Published:** 2020-08-03

**Authors:** Matthew J. McBride, Nazar Mashtalir, Evan B. Winter, Hai T. Dao, Martin Filipovski, Andrew R. D’Avino, Hyuk-Soo Seo, Neil T. Umbreit, Roodolph St. Pierre, Alfredo M. Valencia, Kristin Qian, Hayley J. Zullow, Jacob D. Jaffe, Sirano Dhe-Paganon, Tom W. Muir, Cigall Kadoch

**Affiliations:** 1 Department of Pediatric Oncology, Dana-Farber Cancer Institute and Harvard Medical School, Boston, MA USA; 2 Broad Institute of MIT and Harvard, Cambridge, MA USA; 3 Program in Chemical Biology, Harvard University, Cambridge, MA USA; 4 Department of Chemistry, Princeton University, Princeton, NJ USA; 5 Department of Cancer Biology, Dana-Farber Cancer Institute, Boston, MA USA; 6 Department of Cell Biology, Harvard Medical School, Boston, MA USA; 7 Biological and Biomedical Sciences Program, Harvard Medical School, Boston, MA USA

**Keywords:** Chromatin remodelling, Cancer, Transcription

## Abstract

Interactions between chromatin-associated proteins and the histone landscape play major roles in dictating genome topology and gene expression. Cancer-specific fusion oncoproteins, which display unique chromatin localization patterns, often lack classical DNA-binding domains, presenting challenges in identifying mechanisms governing their site-specific chromatin targeting and function. Here we identify a minimal region of the human SS18-SSX fusion oncoprotein (the hallmark driver of synovial sarcoma) that mediates a direct interaction between the mSWI/SNF complex and the nucleosome acidic patch. This binding results in altered mSWI/SNF composition and nucleosome engagement, driving cancer-specific mSWI/SNF complex targeting and gene expression. Furthermore, the C-terminal region of SSX confers preferential affinity to repressed, H2AK119Ub-marked nucleosomes, underlying the selective targeting to polycomb-marked genomic regions and synovial sarcoma–specific dependency on PRC1 function. Together, our results describe a functional interplay between a key nucleosome binding hub and a histone modification that underlies the disease-specific recruitment of a major chromatin remodeling complex.

## Abstract (web summary)

A combination of cell-based and biochemical approaches reveals how oncogenic fusion protein SS18-SSX directs BAF complexes to H2AK119Ub-modified nucleosomes to remodel chromatin at cancer-specific gene targets.

## Main

A synchronous combination of histone reader domains, chromatin complex conformations, DNA-binding transcription factors (TFs), and other features are required to orchestrate the appropriate targeting of chromatin regulatory machinery in eukaryotic cells. Chromatin reader proteins play critical roles in mediating the engagement of regulatory proteins and protein complexes to specific features of nucleosomal architecture, often to facilitate site-specific catalytic activities. These include bromodomains, which recognize acetylated lysines^1^, plant homeodomains (PHD), which recognize methylation and crotonylatation of histone tails^2,3^, and the increasingly appreciated nucleosome acidic patch interacting domains of SNF2 helicase-based chromatin remodeling complexes^4–6,7^. In parallel, TFs recognize their cognate DNA motifs genome-wide, and, when bound to other proteins or protein complexes, such as chromatin remodeling complexes, can direct their global positioning on chromatin to achieve cell-, tissue- and cancer-specific gene expression programs. For example, TFs have been shown to tether transiently to the surfaces of mammalian SWI/SNF (BAF) ATP-dependent chromatin remodeling complexes to globally reposition them to sites enriched for specific TF DNA-binding motifs^8,9^. Importantly, the results of recent large-scale human genetic sequencing studies indicate that perturbations across each of the above classes of chromatin-bound factors represent frequent and recurrent events in human cancer^10–12^, intellectual disability^13^ and other disorders, with mutations ranging from point mutations and deletions to fusion proteins that alter target engagement and the activity of chromatin regulatory complexes on the genome^14–16^.

It has remained elusive, however, how nuclear fusion oncoproteins that lack canonical TF DNA-binding or recognizable chromatin reader domains yield altered, region-specific targeting of chromatin regulatory proteins and protein complexes. For example, the SS18-SSX fusion oncoprotein involving the BAF complex subunit, SS18, and 78 amino acids of one of the SSX (synovial sarcoma X breakpoint) proteins normally expressed only in testes^17–20^, is a hallmark to 100% of cases of synovial sarcoma (SS). Incorporation of SS18-SSX into BAF complexes causes biochemical changes, such as destabilization of the SMARCB1 (BAF47) subunit, and results in de novo BAF complex targeting to a highly cancer-specific set of sites, particularly broad, polycomb-repressed regions at which polycomb complex occupancy is reduced and gene expression is activated^15,21^. Although some studies have suggested SSX interactions with chromatin-associated factors^22^, the mechanism by which the site-specific binding and unique biochemical properties are achieved remains largely unknown.

Here, we elucidate the mechanism by which the SS18-SSX oncogenic fusion protein engages with chromatin and directs BAF chromatin remodeling complexes to specialized target sites. Specifically, we find that SSX contains a basic region that directly binds the nucleosome acidic patch, altering BAF complex subunit configuration and activity. Furthermore, SSX-nucleosome binding is augmented by the presence of ubiquitylated histone H2A (H2AK119Ub) on nucleosomes, preferential recognition of which requires a second, conserved region of SSX. These dual reader-like features of SSX underlie the highly disease-specific chromatin remodeling complex targeting, gene expression and functional dependencies in SS. Collectively, our studies reveal a novel mechanism of chromatin localization with important implications in both disease biology and therapeutic discovery.

## Results

### SS18-SSX-bound BAF complexes bind chromatin with uniquely high affinity

**Fig. 1:**
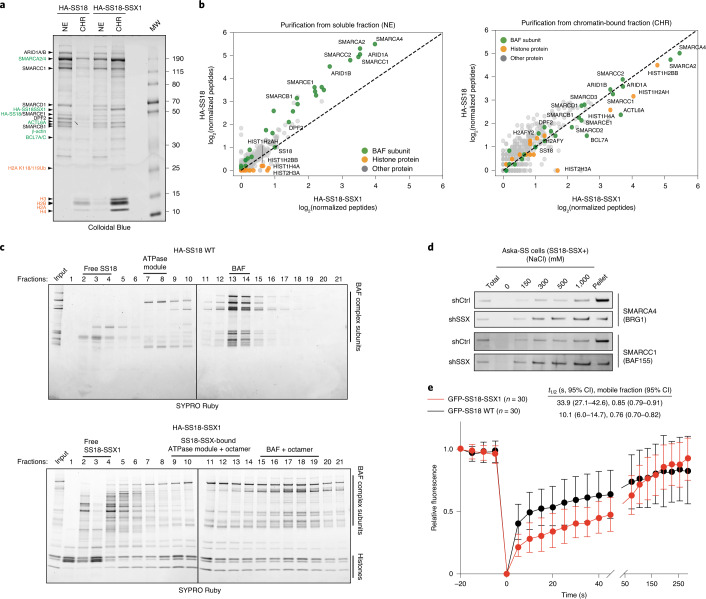
SS18-SSX-containing BAF complexes exhibit increased affinity for chromatin. **a**, Colloidal blue staining of WT BAF complexes (from HA-SS18 WT-expressing 293T cells) and SS18-SSX-contaning BAF complexes (from HA-SS18-SSX1-expressing cells), purified from soluble NE and CHR fractions. Equal amounts (by volume) of nuclei were fractionated, as described in the Methods. BAF complex subunits (black, with ATPase module components highlighted in green) and histone proteins (orange) are identified at left. **b**, MS spectral counts for BAF complex subunits (green) and histone proteins (orange) from HA-SS18 WT and HA-SS18-SSX purifications in **a**. Peptide counts are log_2_-normalized to bait (SS18 peptides). **c**, SDS–polyacrylamide gel electrophoresis (SDS–PAGE) of 10–30% glycerol gradient sedimentation performed on purified HA-SS18 WT (from NE) and HA-SS18-SSX1 (from CHR) fractions from HEK293T cells. BAF complex subunits and histone proteins are indicated. SYPRO Ruby staining was used for visualization. **d**, Immunoblot of SMARCA4 and SMARCC1 performed on Aska SS cells in shCtrl (control, non-targeting hairpin shRNA) and shSSX (shRNA targeted to SSX) conditions following differential salt extraction (0–1,000 mM NaCl). **e**, FRAP studies performed on HEK293T cells expressing either GFP-SS18 WT or GFP-SS18-SSX1. Recovery kinetics were recorded and the recovery half times were 10.1 and 33.9 s for GFP-SS18 WT and GFP-SS18-SSX1, respectively. Values represent mean ± s.d. of *n* = 30 cells per condition collected from two biological replicates. *t*_1/2_, 95% confidence interval (CI) and mobile fraction values were determined by fitting a curve to the data using nonlinear regression. Data for **a** and **b** are presented in Supplementary Table 1. Uncropped gel images for **a**, **c** and **d** are presented in Supplementary Fig. 1 and are available as Source data. Raw binding data for **e** are available as Source data. Source data

**Extended Data Fig. 1:**
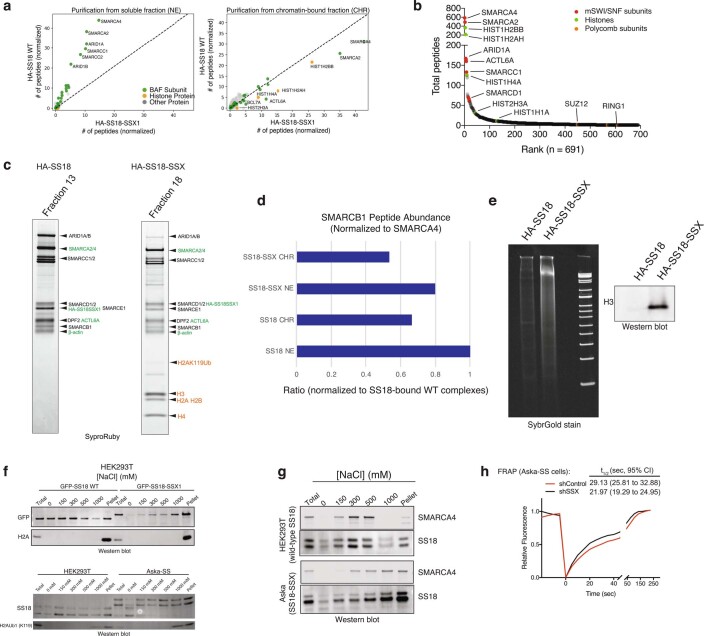
SS18-SSX-containing BAF complexes exhibit high-affinity interactions with histones and longer residency times on chromatin. **a**, MS spectral counts for BAF complex subunits and histone proteins from HA-SS18 WT and HA-SS18-SSX purifications from soluble nuclear extract NE and CHR fractions from Fig. 1a. Total number of peptides (normalized to bait, SS18), are shown. **b**, Ranked peptides captured in HA-SS18-SSX purification (chromatin-bound fraction). Red, mSWI/SNF complex subunits. Green, histones. Orange, members of PRC1 and PRC2 complexes, shown for comparison. See also Supplementary Table 1. **c**, SYPRO Ruby staining indicating identified proteins from Fig.1c in Fraction 13 (HA-SS18 WT) and Fraction 18 (HA-SS18-SSX).d, SMARCB1 peptide abundance (normalized to SMARCA4) and relative to SS18 WT-bound complexes (soluble NE fraction). NE, nuclear extract; CHR, chromatin-bound fraction. **e**, (left) Cyber-gold staining of complexes purified from untreated (no benzonase) nuclear extracts isolated via ammonium sulfate extraction. (right) H3 immunoblot reveals prominent histone binding in HA-SS18-SSX-bound complexes but not in HA-SS18 WT-bound complexes. **f**, (Top) Immunoblot for GFP and H2A performed on HEK293T cells infected with either GFP-SS18 WT or GFP-SS18-SSX following differential salt extraction (0-1000 mM NaCl).(Bottom) Immunoblot for SS18 and H2AK119Ub performed on HEK293T cells (naive) and Aska-SS cells following differential salt extraction (0-1000 mM NaCl) experiments. **g**, Immunoblot for SMARCA4 and SS18 performed HEK293T cells or Aska SS cells (SS18-SSX + ) following differential salt extraction (0-1000 mM NaCl). **h**, FRAP experiments performed in the Aska SS cell line modified to express BRG1 (SMARCA4)-Halo. Aska-SS cells were treated witih either shControl shRNA hairpin or shSSX (targeting the SS18-SSX fusion). Values represent mean of n = 30 cells per condition collected from two biological replicates and t_1/2_ and 95% CI values were determined by fitting a curve to the data using nonlinear regression. Uncropped gel images for all relevant panels are presented in Supplementary Figure 1. Data for panels 1d, 1e, and 1 h are available as source data.

Recent studies have indicated that SS18-SSX-bound BAF complexes have specialized biochemical and chromatin localization properties^15,16^. We sought to explore the underlying molecular recognition mechanisms driving these associations and activities. To this end, we expressed human influenza hemagglutinin (HA)-tagged versions of either wild-type (WT) SS18 or SS18-SSX in HEK293T cells and performed BAF complex purifications from soluble nuclear extract (NE) and nuclease-treated solubilized chromatin (CHR) (Fig. 1a). Strikingly, fusion oncoprotein SS18-SSX-bound BAF complexes preferentially eluted in the CHR material, in contrast to WT complexes, which eluted nearly completely in the soluble NE material, as expected from previous studies examining WT (and other loss-of-function mutant variants of) BAF complexes^10,23^. Importantly, SS18-SSX-bound complexes captured near-stoichiometric amounts of core histone proteins H2A, H2B, H3 and H4 (Fig. 1a). We next subjected these complexes to mass spectrometric (MS) analyses and found selective co-enrichment of histone peptides with HA-SS18-SSX, but not with HA-WT SS18 in the chromatin-bound fractions (Fig. 1b and Extended Data Fig. 1a,b). Notably, we captured peptides corresponding to the H2AK119Ub mark only in the purifications of SS18-SSX-bound complexes but not in SS18 WT complexes, in agreement with the visualization of this mark upon colloidal blue staining (Fig. 1a and Supplementary Table 1). SS18-SSX purifications most substantially enriched for ATPase subunits SMARCA4 and SMARCA2, BCL7A, ACTL6A and β-actin, consistent with the fact that SS18 is part of the ATPase module of mSWI/SNF complexes^23^, while core module components, particularly SMARCB1, were less enriched compared to WT SS18 purifications (Fig. 1b, Extended Data Fig. 1c,d and Supplementary Table 1). We did not detect binding to polycomb repressive complex 1 (PRC1) components, as has been suggested previously^22^ (Extended Data Fig. 1b).

Purification of SS18-SSX-bound complexes followed by density sedimentation using 10–30% glycerol gradients revealed larger-sized fusion-containing BAF complexes migrating in fractions 15–19 compared to WT SS18-bound complexes in fractions 13–14, as expected^23^, suggesting high-affinity, stable binding of SS18-SSX-bound BAF complexes to the full histone octamer (Fig. 1c and Extended Data Fig. 1e). In addition, we observed histones bound to the ATPase module components in isolation as well as to free SS18-SSX in fractions 9–13 and 2–4, respectively. These results suggest that fusion-containing BAF complexes bind nucleosomes more strongly than WT SS18-containing BAF complexes, which did not co-purify with nucleosomes in ultracentrifugation experiments. Finally, to determine the relative chromatin affinities of WT BAF complexes versus SS18-SSX-containing BAF complexes, we performed differential salt extraction in both SS cell lines and HEK293T cells expressing SS18-SSX (Fig. 1d and Extended Data Fig. 1f,g). We observed normal extraction profiles for WT complexes (elution at 300–500 mM NaCl), consistent with previous findings^24,25^; however, fusion-containing complexes remained insoluble in up to 1 M NaCl. In support of these findings, fluorescence recovery after photobleaching (FRAP) experiments in HEK293T cells infected with either GFP-SS18 or GFP-SS18-SSX revealed substantially increased chromatin residency times for SS18-SSX-bound BAF complexes (Fig. 1e and Extended Data Fig. 1h). Taken together, these findings indicate an unexpected, uniquely high-affinity conjugation of SS18-SSX-bound BAF complexes to nucleosomes, suggesting this as a feature that may underlie the site-specific targeting of SS18-SSX complexes on chromatin.

### A minimal 34-amino-acid region of SSX is necessary and sufficient for direct binding to repressive nucleosomes and SS18-SSX-mediated oncogenic functions

**Fig. 2:**
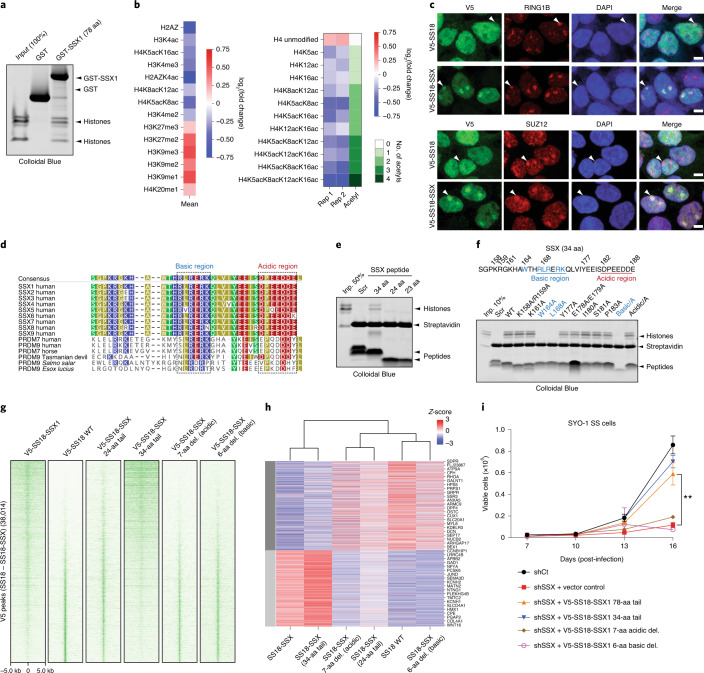
Conserved basic and acidic regions within a minimal SSX domain are necessary and sufficient for nucleosome binding and BAF complex recruitment and activity. **a**, Glutathione *S*-transferase (GST; control) and GST-SSX1 (78 aa) purified recombinant proteins incubated with mammalian mononucleosomes (purified by MNase digestion) were captured using glutathione resin and visualized using Colloidal Blue stain. **b**, Quantitative MS analysis of maltose binding protein (MBP) pull-down experiments using the MBP-SSX 78-aa protein and endogenous mammalian nucleosomes purified using MNase digestion from 293T cells. The log_2_(fold change (FC)) is calculated relative to the input sample. Red, enriched; blue, depleted. Raw data are provided in Supplementary Table 2. **c**, IF analysis of V5-tagged SS18 and SS18-SSX relative to RING1B and SUZ12 in 293T cells. Arrowheads indicate Barr bodies. Scale bars, 5 μm. **d**, SSX1 protein sequence alignment across species and compared to related PRDM7 and PRDM9 proteins. Highly conserved basic (blue) and acidic (red) regions are indicated. **e**, Pull-down experiments with biotinylated SSX peptides (scrambled (aa 155–188), SSX 34 aa (aa 155–188), SSX 24 aa (aa 164–188) and SSX 23 aa (aa 165–188) incubated with mammalian mononucleosomes and visualized with Colloidal Blue. **f**, Pull-down experiments of N-terminally biotinylated SSX peptides, including scrambled control, WT and mutant variants (single alanine substitutions as well as regional substitutions; that is, basic-A, basic region RLRERK → AAAAAA; acidic-A, acidic region DPEEDDE → AAAAAAA), incubated with mammalian mononucleosomes and visualized with Colloidal Blue. **g**, ChIP-seq density heatmaps reflecting the chromatin occupancy of V5-SS18-SSX1, V5-SS18, V5-SS18-SSX (24 aa) and V5-SS18-SSX (34 aa) over all V5 peaks (38,014 total peaks). **h**, Heatmap reflecting the top 5% upregulated and downregulated genes (*Z*-score) by RNA-seq for each condition. **i**, Proliferation of SYO1 SS cells infected with either control hairpin (shCt) or shSSX with overexpression of empty vector control, SS18-SSX 78 aa or SS18-SSX 34 aa variants. Error bars represent mean ± s.d. of *n* = 3 independent experimental replicates; **P < 0.01 determined from a two-tailed *t*-test. Data for **i** are available as Source data. Uncropped gel images for **a**, **e** and **f** are presented in Supplementary Fig. 1. Source data

**Extended Data Fig. 2:**
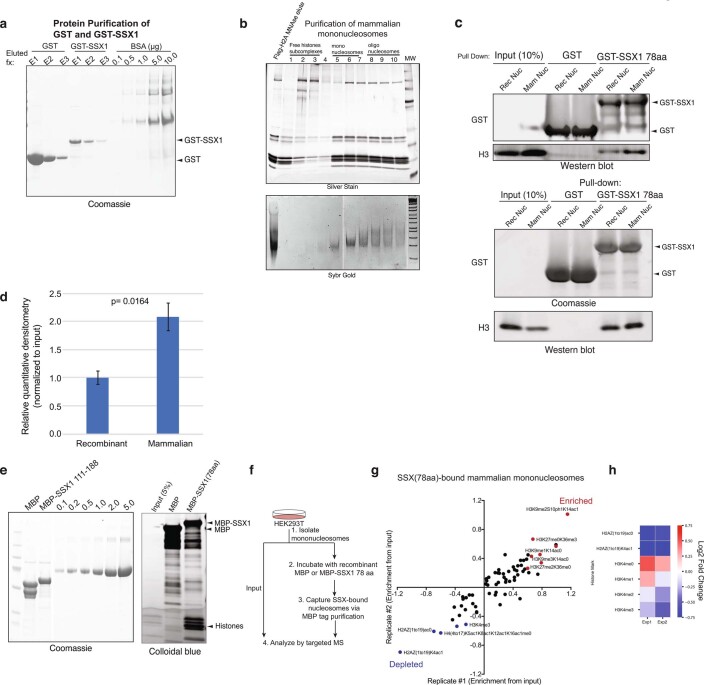
The SSX 78aa protein binds mononucleosomes, with preference for nucleosomes decorated with repressive histone modifications. **a**, Coomassie-stained gel of recombinantly-purified GST, GST-SSX (78aa) proteins, run next to BSA protein as control. **b**, Purification of mammalian mononucleosomes from HEK293T cells using MNase digestion. **c**, Incubation of GST or GST-SSX (78aa) with either recombinant or mammalian mononucleosomes, resolved by immunoblot for GST and histone H3 or Coomassie and histone H3. Two representative experiments are shown. **d**, Quantitative densitometry of histone H3 normalized to input reflecting GST-SSX 78aa preferential binding to mammalian mononucleosomes (prepared via MNase digestion in HEK293T cells) versus recombinant, unmodified nucleosomes. Bars represent averages of n = 3 independent experiments, error bars represent mean ± s.d.; p-value= 0.0164 using two-tailed Student’s t-test. **e**, Purification of MBP and MBP-SSX (78aa) proteins for targeted, quantitative histone mass-spectrometry. **f**, Schematic for targeted MS experiments. **g**, Enrichment of SSX-bound histone peptides, over input. Enriched and depleted proteins are shown in red and blue, respectively. Quantitative histone mass spectrometry performed on MBP-SSX1 (versus MBP control) incubated with pooled mononucleosomes isolated from HEK293T cells via MNase digestion. Experiment performed in n = 2 replicates. See also Supplementary Table 2. **h**, Heatmap reflecting enrichment or depletion of selected histone marks, including H2AZ and H3K4 methylation states. Scale= log2FC (normalized to input), blue (negatively enriched), red (positively enriched). See also Supplementary Table 2. Uncropped gel images for all relevant panels are presented in Supplementary Figure 1. Data for panels 2d, 2g-2h are available as source data.

**Extended Data Fig. 3:**
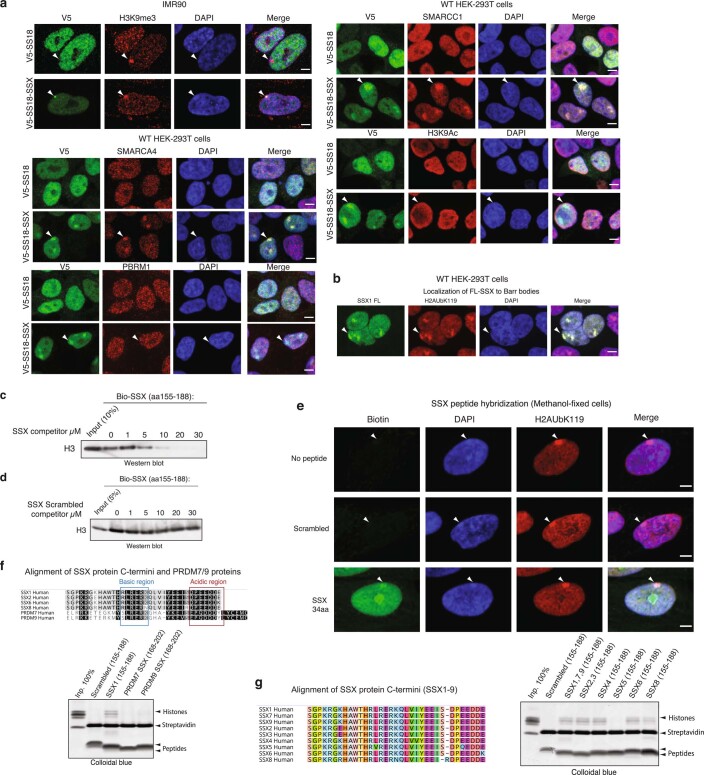
Nucleosome binding and nuclear localization properties of SS18-SSX and SSX variants. **a**, Immunofluorescence imaging performed on IMR90 fibroblasts and HEK293T cells infected with either V5-SS18-SSX or V5-SS18. Visualized in red for H3K9me3, SMARCA4, PBRM1, SMARCC1, H3K9Ac across experiments. DAPI is shown as nuclear stain and merged images are provided with scale bars, Scale bar, 5μm. **b**, IF-based localization of SSX1 FL (1-188aa) in fibroblasts. H2AK119Ub, DAPI counterstain, and merged images are shown. Scale bar, 5μm. **c**, Peptide competition experiment using Biotinylated SSX peptide (aa 155-188) and unlabeled SSX (aa 155-188). Visualization for Histone H3 using immunoblot. **d**, Peptide competition experiment using Biotinylated SSX peptide (aa 155-188) and Scrambled control SSX peptide (aa 155-188). Visualization for Histone H3 using immunoblot. **e**, SSX peptide hybridization experiments performed on methanol-fixed cells. Streptavidin (SA) used for visualization of biotinylated SSX peptides, H2AK119Ub for Barr bodies. DAPI counterstain and merged images shown. Scale bar, 5μm. **f**, Top, conservation analysis among SSX and PRDM7 and PRDM9 human protein regions. Bottom, peptide pull-down experiments with recombinant nucleosomes performed with Scrambled control SSX1, SSX1, PRDM7, PRDM9. Visualization by colloidal blue staining. **g**, Left, alignment of SSX proteins (SSX1-9). Right, peptide pull-down experiments with recombinant nucleosomes performed with aa 155-188 of SSX family members. Visualization by colloidal blue staining. Uncropped gel images are presented in Supplementary Figure 1.

We next sought to determine whether the 78 residues of SSX in isolation (not fused to the SS18 subunit and hence not part of BAF complexes) could directly bind nucleosomes and could be responsible for conferring the unique affinity and nucleosome-binding properties of the SS18-SSX fusion protein. Indeed, pull-down experiments revealed that the C-terminal 78 residues of SSX (amino acids (aa) 111–188) were sufficient for its nucleosomal interactions (Fig. 2a and Extended Data Fig. 2a,b). In addition, we found that binding to mammalian nucleosomes (purified via MNase digestion of HEK293T cell chromatin and hence representing the diverse array of histone variants and modifications) was stronger than binding to recombinant, unmodified nucleosomes (Extended Data Fig. 2c,d), indicating that a mammalian histone modification might provide added affinity and site specificity. In agreement with this, targeted quantitative MS analysis of SSX-bound mammalian nucleosomes (pooled, purified by MNase digestion from HEK293T cells, containing the full diversity of histone marks) revealed strong enrichment of nucleosomes decorated with known repressive histone marks and depletion of nucleosomes with known activation marks (Fig. 2b, Extended Data Fig. 2e–h and Supplementary Table 2). For example, we detected enrichment of nucleosomes decorated with repressive marks such as H3K27me3 and H3K9me3, and depletion of nucleosomes decorated with activating marks such as H4 lysine acetylation and H3K4me2/3 (while nucleosomes containing unmodified H4 and H3 were enriched) in these SSX–mammalian nucleosome binding experiments. Further, immunofluorescence (IF) analyses revealed strong co-localization of SS18-SSX as well as SSX in isolation (SSX aa 1–188, as expressed in testes) to Barr bodies marked with repressive PRC1 and PRC2 complexes and their marks (Fig. 2c and Extended Data Fig. 3a,b).

SSX-like protein sequences are only found in mammalian SSX family proteins (human SSX1-9) and members of the vertebrate-specific PRDM7 and PRDM9 methyltransferases. We identified a 34-aa region of SSX (SSX aa 155–188) that is highly conserved across vertebrate species of SSX (putative PFAM SSXRD domain) and is similar to that of PRDM7 and PRDM9 proteins (Fig. 2d). Pull-down experiments using biotinylated peptides corresponding to this region indicated it was sufficient for SSX-nucleosome binding, while shorter 23- (SSX aa 166–188) and 24- (SSX aa 165–188) residue peptides (lacking the W164 residue) failed to do so (Fig. 2e). This SSX-nucleosome interaction was specific, as biotin pull-downs were outcompeted by the addition of unlabeled SSX 34-residue peptide and could not be competed by a scrambled control peptide corresponding to the same SSX 34-aa region (Extended Data Fig. 3c,d).

To define whether SSX 34-residue peptide could be used as a probe for repressive Barr bodies or polycomb bodies in cells, we implemented a peptide hybridization approach performed on methanol-fixed (non-crosslinked) IMR90 fibroblasts incubated with biotinylated SSX peptides and subsequently co-stained with the Barr body marker H2AK119Ub. We observed clear labeling of Barr bodies suggesting an innate ability of the SSX 34-residue region to selectively localize to repressed chromatin regions (Extended Data Fig. 3e). The 34-aa regions corresponding to most human SSX proteins exhibited interactions with nucleosomes, while shorter SSX-like sequences found in in PRDM7 or PRDM9 proteins lacking the W164 and first R residues of the basic region (R167) failed to do so, suggesting a newly evolved, mammalian-specific function of this full protein region (Extended Data Fig. 3f,g). Finally, to identify residues important for nucleosome binding, we designed a library of 34-residue SSX peptides containing alanine substitutions in either single conserved residues or alanine substitutions across the full basic and acidic regions. Importantly, these experiments revealed that the core residues of the 6-aa basic region of SSX (RLRERK) were required, as single-residue and full region alanine substitutions in this region completely abrogated SSX-nucleosome binding (Fig. 2f).

**Extended Data Fig. 4:**
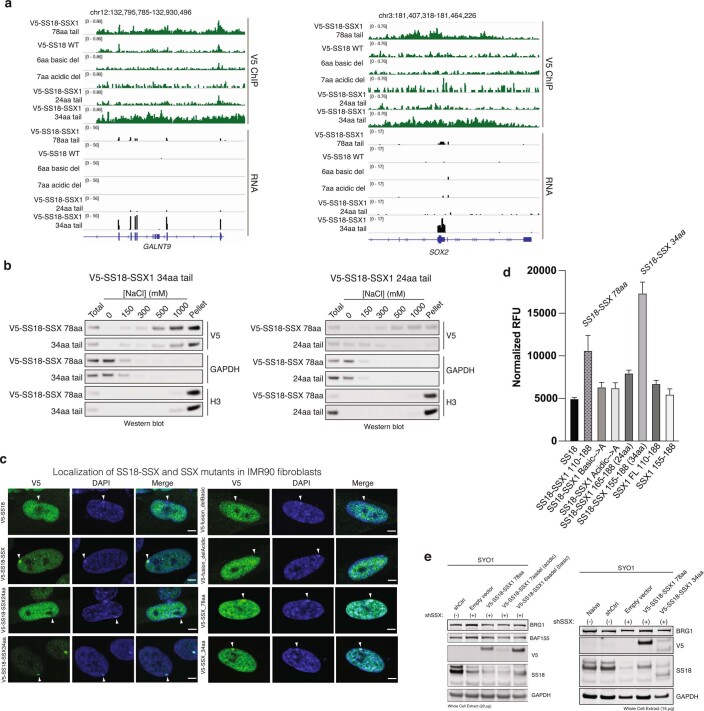
Defining a minimal 34-aa SSX region responsible for chromatin engagement and oncogenic gene expression. **a**, Additional representative V5 ChIP-seq and RNA-seq tracks, here shown at the GALNT9 and SOX2 loci. **b**, Differential salt experiments ([0-1000 mM NaCl]) performed on HEK293T cells infected with either SS18-SSX 34aa versus SS18-SSX 24aa. Immunoblots for V5 as well as GAPDH and H3 (controls) are shown. **c**, Immunofluorescence imaging of IMR90 fibroblasts infected with SS18 and SS18-SSX variants, as indicated, and stained for V5 (SS18-SSX or SSX variant) and DAPI; merged images are shown. Localization to H2AUb119-high sites (Barr bodies) is highlighted. Scale bar=5μm. **d**, Beta-gal senescence assay performed on IMR90 cells infected with WT SS18, SS18-SSX and SSX FL and 78aa variants, as indicated. Averaged normalized RFUs are shown (bar graph indicates mean RFU across n = 3 independent experiments; error bars represent mean ± s.d. **e**, SYO1 synovial sarcoma cells were treated with either shCtrl (control hairpin) or shSSX (shRNA targeting SSX) to reduce levels of endogenous fusion, followed by rescue of SS18-SSX WT and mutant variants or empty vector control. Proliferation was evaluated over 16 days (see also Fig. 2i). Uncropped gel images are presented in Supplementary Figure 1. Data for panel d are available as source data.

To determine whether these minimal regions were sufficient for the genome-wide targeting of fully-formed, endogenous SS18-SSX-containing BAF complexes in cells, we expressed either WT SS18, SS18-SSX or SS18 fused to a range of mutant SSX variants by lentiviral infection in CRL7250 human fibroblasts. ChIP-seq experiments revealed that the 34-aa SSX tail fused to SS18 was sufficient to achieve SS18-SSX targeting, while the 24-aa fusion was unable to do so (Fig. 2g and Extended Data Fig. 4a). Notably, deletion of either the basic or the acidic conserved regions of SSX resulted in complete loss of oncogenic fusion complex targeting, suggesting that both of these regions are required for SS18-SSX-specific properties. These findings were consistent with biochemical results suggesting that the full 34-aa tail is needed to confer tight affinity of SS18-SSX to chromatin in cells (Extended Data Fig. 4b). Importantly, these changes in chromatin targeting resulted in corresponding changes in gene expression by RNA-seq, as evidenced by clustering of the transcriptional profiles of the 34-residue tail fusion with the full SS18-SSX fusion (78-aa fusion tail), while deletion of either basic or acidic conserved regions or 24-aa SSX tail variants clustered with SS18 WT gene expression profiles (Fig. 2h). These findings were further corroborated using IF for SS18-SSX Barr body localization (Extended Data Fig. 4c) as well as β-galactosidase senescence assays in IMR90 fibroblasts performed across SS18-SSX and SSX (alone) variants (Extended Data Fig. 4d). Finally, both SS18-SSX 78-aa and 34-aa minimal fusions rescued proliferation in SS cell lines that are well-established to be dependent on the function of SS18-SSX and bearing shRNA-mediated knockdown (KD) of the endogenous SS18-SSX fusion. Taken together, these data suggest that the 34-aa minimal region of SSX that contains the conserved basic and acidic regions is responsible for the maintenance of oncogenic gene expression and proliferation in SS cell lines driven by the SS18-SSX fusion oncoprotein (Fig. 2i and Extended Data Fig. 4e).

### An RLR motif within the SSX basic region competes with SMARCB1 for nucleosome acidic patch binding

**Fig. 3:**
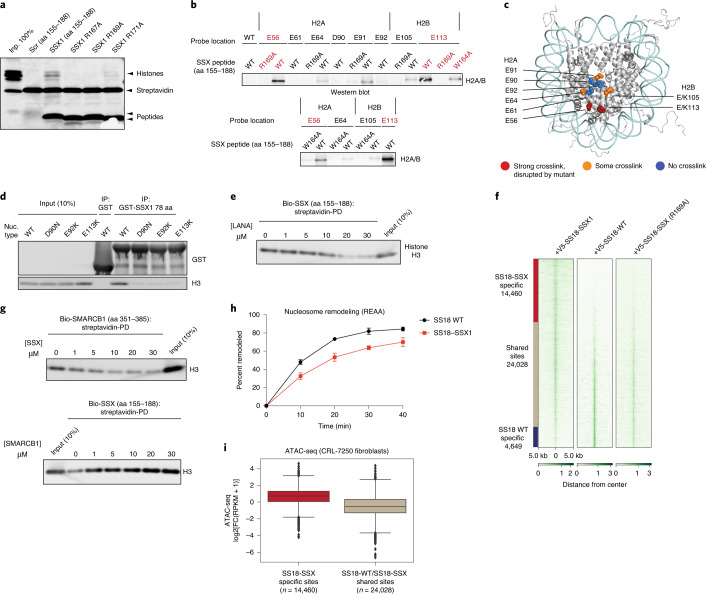
The SSX basic region outcompetes the SMARCB1 C-terminal α-helical domain for nucleosome acidic patch binding. **a**, Incubation of biotinylated SSX peptides (aa 155–188) in either WT or RLR motif-mutant forms (R167A, R169A, R171A) with nucleosomes. Proteins are visualized by Colloidal Blue staining. **b**, Western blots of photocrosslinking assays performed with reactive diazirine probes localized to the indicated nucleosome acidic patch residues. Red labels indicate strongest binding to H2A E56 and H2B E113. **c**, SSX binding sites mapped on the nucleosome structure (PDB 1KX5). Acidic patch crosslinked sites are labeled. **d**, Pull-down assays of the GST-SSX 78-aa tail with either WT or the indicated acidic patch mutant nucleosomes (D90N, E92K and E113K). Binding is visualized by histone H3 immunoblotting. **e**, LANA peptide competition experiment with SSX 34-aa biotinylated peptide bound to nucleosomes. **f**, V5 ChIP-seq heatmap reflecting genome-wide localization of V5-tagged SS18-SSX, SS18 WT and SS18-SSX RLR → RLA (R169A) mutant in CRL7250 fibroblasts. **g**, Reciprocal competition experiments performed with either SMARCB1 C-terminal α-helical domain bound to nucleosomes or SSX 34 aa bound to nucleosomes and competed with the indicated peptide. Binding is visualized by histone H3 immunoblotting. **h**, REAA nucleosome remodeling assay performed with BAF complexes containing either WT SS18 or SS18-SSX. A 0–40-min time course was performed at 37 °C. BAF complex capture was performed using ARID1A IP. Error bars represent mean ± s.d. of *n* = 3 experimental replicates. **i**, Box and whisker plot depicting ATAC-seq DNA accessibility (log_2_FC(RPKM + 1) performed in CRL7250 fibroblasts over SS18-SSX-specific sites and SS18 WT SS18-SSX shared sites, as defined in **f**. Boxes represent the interquartile range (IQR), the horizontal bar represents the median, and the minima and maxima show 1.5 × IQR from the box. Raw data are provided in the Source data. Uncropped gel images for **a**, **d**, **e** and **g** are presented in Supplementary Fig. 1. Source data

**Extended Data Fig. 5:**
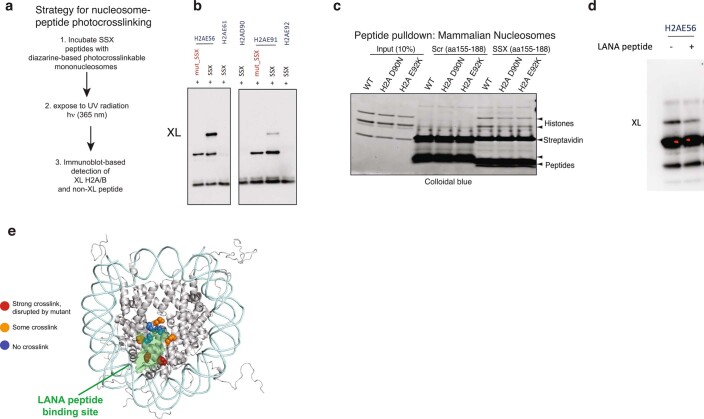
The SSX basic region and SMARCB1 C-terminal alpha helical domain compete for nucleosome acidic patch binding. **a**, Schematic outlining strategy for nucleosome-peptide photocrosslinking. **b**, Additional (replicate) photocrosslinking experiments performed with reactive diazirine probes localized throughout the nucleosome acidic patch region indicate strongest binding to H2A E56 and H2B E113 residues, weaker binding to H2A E91, and no binding to E61, E92, and D90 residues. Experimental conditions: 0.3uM mononucleosomes, 3uM SSX, 150 mM KCl. **c**, Pull-down experiments performed with either Scrambled or SSX 34aa peptides (biotinylated) incubated with mammalian mononucleosomes prepared from cells infected with WT H2A, or H2AD90N, H2A E92K mutant variants. **d**, Photocrosslinking experiments performed with SSX 34aa peptide incubated with nucleosomes modified at the H2A E56 residue, with and without LANA peptide competition. **e**, Model indicating docking of solved LANA peptide-nucleosome binding region and SSX peptide crosslinking sites in the nucleosome acidic patch. Interacting residues from photocrosslinking experiments are highlighted. Uncropped gel images for all relevant panels are presented in Supplementary Figure 1.

**Extended Data Fig. 6:**
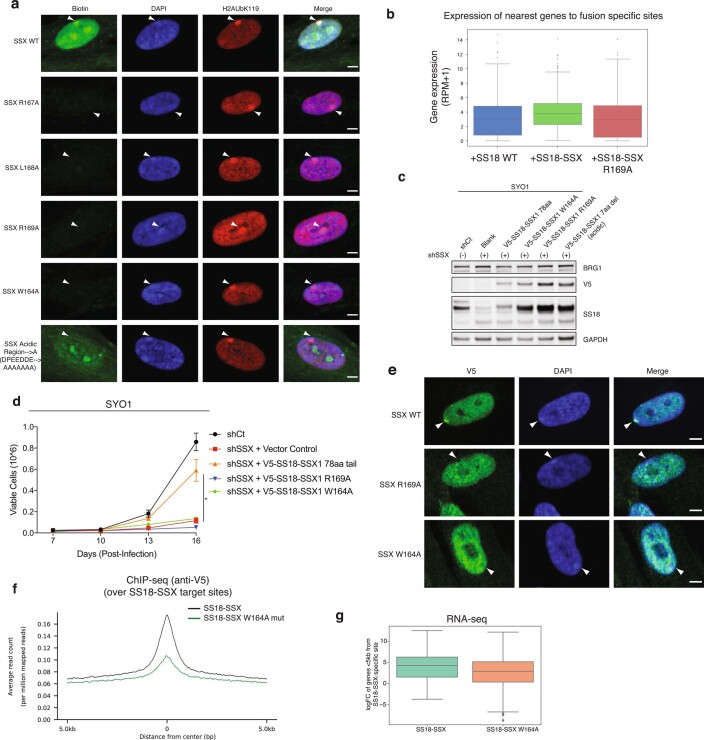
Mutations in the basic region of SSX affect the targeting and function of SS18-SSX-containing BAF complexes. **a**, Peptide hybridization of IMR90 cells using SSX and mutant basic region mutant peptides. Arrows indicate positions of the Barr bodies. Scale bar=5μm. **b**, Gene expression changes across each SS18 WT and SS18-SSX variant conditions from Fig. 3g. **c**, Immunoblot performed on whole-cell extracts (RIPA extraction) from SYO1 cells treated with either shCtrl or shSSX and infected with either empty vector or SS18-SSX variants, used in proliferation experiments in Extended Data Fig. 6d. **d**, Proliferative rescue experiments performed in SYO1 SS cell line treated with shSSX and rescued with either vector control, SS18-SSX or SS18-SSX (R169A or W164A) variants. Error bars represent mean ± s.d. of n = 3 independent experimental replicates; * p < 0.05 determined from a two-tailed t-test. **e**, Peptide hybridization of IMR90 cells using SSX and mutant basic region mutant (W164A and R169A) peptides. Arrows indicate positions of the Barr bodies. Scale bar= 5μm. **f**, ChIP-seq studies (anti-V5) performed in CRL7250 cells infected with either SS18-SSX or SS18-SSX W164A mutant, mapped as summary plot over SS18-SSX target sites. **g**, RNA-seq (gene expression), box and whisker plots indicating average expression in SS18-SSX versus SS18-SSX W164A mutant conditions. Uncropped gel images for all relevant panels are presented in Supplementary Figure 1. Data for panel d are available as source data.

Using systematic mutagenesis on the SSX 34-residue region, we found that single-residue perturbations to the basic region, which includes a Kaposi’s sarcoma-associated herpesvirus (KSHV) LANA (latency-associated nuclear antigen)-like RLR motif, resulted in complete loss of nucleosome binding (Fig. 3a). These data suggested that this highly basic region binds directly to the H2A-H2B acidic patch of the nucleosome. To identify the specific sites involved in acidic patch engagement, we introduced reactive diazirine probes at various residues within the nucleosome acidic patch and performed photocrosslinking studies^6^ with SSX 34-residue peptides (Fig. 3b and Extended Data Fig. 5a,b). Histone-SSX crosslinks were identified at several positions across the extended acidic patch region, most prominently at positions H2A E56 and H2B E113, which, importantly, were substantially reduced when key RLR basic residues in SSX were mutated (Fig. 3b,c). To probe this further, we assembled nucleosomes containing H2A mutant variants D90N, E92K and E113K, which disrupt the integrity of the acidic patch for GST-SSX pull-down experiments. These experiments showed near complete loss of SSX binding to acidic patch-mutant nucleosomes, indicating the importance of this highly conserved and important docking site for the SSX-chromatin interaction (Fig. 3d (homotypic) and Extended Data Fig. 5c (heterotypic)). These results were further corroborated by the fact that we observed direct nucleosome binding competition between the LANA peptide and SSX (Fig. 3e and Extended Data Fig. 5d,e), as the LANA peptide is well-established to bind the nucleosome acidic patch^26^. In cells, single-residue mutations within the nucleosome acidic patch binding region of SSX (SSX R169A as well as W164A) resulted in attenuation of SS18-SSX-specific BAF complex chromatin occupancy, recruitment to Barr bodies, gene expression activation and proliferative maintenance in SS cell lines (Fig. 3f and Extended Data Fig. 6a–g). Taken together, these data establish the role for the basic region, specifically the RLR motif, in mediating SS18-SSX-nucleosome binding, in conferring SS18-SSX-containing BAF complex chromatin binding properties, as well as in the maintenance of gene expression and proliferation.

**Extended Data Fig. 7:**
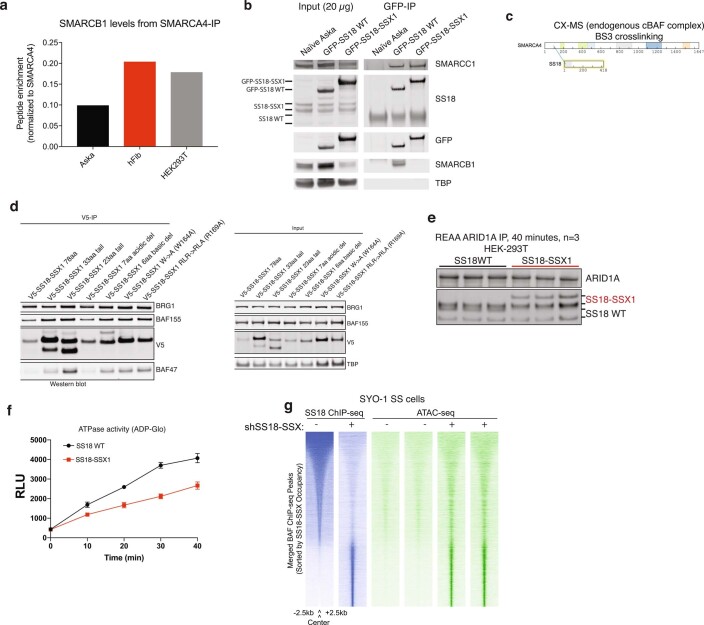
Subunit composition, chromatin binding, and functional properties of SS18-SSX-bound BAF complexes. **a**, SMARCB1 peptide abundance calculated from MS experiments (anti-SMARCA4 (BRG1) IPs) performed in Aska-SS synovial sarcoma cells, human Fibroblasts, and HEK293T cells. Peptide abundance normalized to SMARCA4 abundance. **b**, Input and GFP IPs performed in Aska-SS cells infected with either GFP-SS18 or GFP-SS18-SSX. SMARCC1, SS18, GFP, SMARCB1, and TBP levels are shown. **c**, SS18-SMARCA4 crosslinks detected in CX-MS experiments of intact, fully-formed canonical BAF complexes in Mashtalir et al., Cell 2018. **d**, Immunoblot studies performed on CRL7250 cells infected with SS18-SSX variants indicated. **e**, Immunoblot performed for ARID1A and SS18 on complexes captured via ARID1A, used for nucleosome remodeling and ATPase assays. **f**, ATPase activity calculated by ADP-Glo for SS18 WT- and SS18-SSX-containing BAF complexes. t, 0-40 min time course, n = 3 experimental replicates at each time point; error bars represent mean ± s.d. **g**, ATAC-seq studies performed in SYO1 SS cells in shCtrl and shSSX conditions, mapped over SS18 ChIP-seq. Uncropped gel images for all relevant panels are presented in Supplementary Figure 1. Data for panel f are available as source data.

We have previously demonstrated that, upon SS18-SSX expression and incorporation into BAF complexes, the SMARCB1 (BAF47) subunit of BAF complexes, part of the core module^23^, is destabilized and proteasomally degraded^16^ (see also Extended Data Fig. 7a,b and Supplementary Table 3). Intriguingly, using pull-down competition assays, we found that SSX competed with the recently identified SMARCB1 C-terminal α-helix (aa 351–385) region^7,27^ for nucleosome acidic patch binding (Fig. 3g)^7,27^. However, the reverse was not true, as the SMARCB1 C-terminal α-helix was unable to outcompete SSX from binding the nucleosomes, implicating stronger affinity of SSX compared to SMARCB1 C terminus for nucleosomes. This result, coupled with the positioning of SS18 at the very N terminus of the SMARCA4 subunit within the core module of BAF complexes (defined by CX-MS (Extended Data Fig. 7c), recent BAF complex structural insights^7,27,28^ and assessment of SMARCB1 levels across SS18-SSX mutant conditions (Extended Data Fig. 7d)), suggests the mechanism of decreased BAF complex binding and degradation of SMARCB1 observed in SS cell lines^15,16,29^ may be explained by the dominant, higher-affinity SSX binding to the nucleosome acidic patch and the resulting configurational changes within the BAF core module.

Finally, to evaluate whether SS18-SSX-containing BAF complexes that are tethered to the nucleosome acidic patch via SSX in place of the BAF core module SMARCB1 C-terminal acidic patch binding region^7^ are competent in remodeling, we assessed their activity using restriction enzyme accessibility assays (REAA) on endogenous BAF complexes containing either SS18 WT or SS18-SSX as well as DNA accessibility using an assay for transposase-accessible chromatin using sequencing (ATAC-seq) in both CRL7250 fibroblasts and SS cell lines. Remodeling efficiency and ATPase activity of SS18-SSX-bound BAF complexes were slightly lower than for WT SS18-bound complexes (Fig. 3h and Extended Data Fig. 7e,f); however, this reduced activity was sufficient to enable DNA accessibility over SS18-SSX target sites genome-wide (Fig. 3i and Extended Data Fig. 7g).

Taken together, these data resolve SSX as a nucleosome acidic patch binding ligand fused to SS18, a subunit bound to the N terminus of the BAF complex ATPase subunit (SMARCA4) within the core structural module^7,27^, that dominantly competes for acidic patch binding with BAF core module subunit SMARCB1, resulting in its partial destabilization and degradation. These oncogenic SS18-SSX-containing complexes are still proficient in chromatin remodeling and catalytic activities, resulting in the aberrant activation of normally repressed chromatin regions.

### SSX exhibits preference for H2AK119Ub-marked nucleosomes via its conserved C-terminal acidic region

**Fig. 4:**
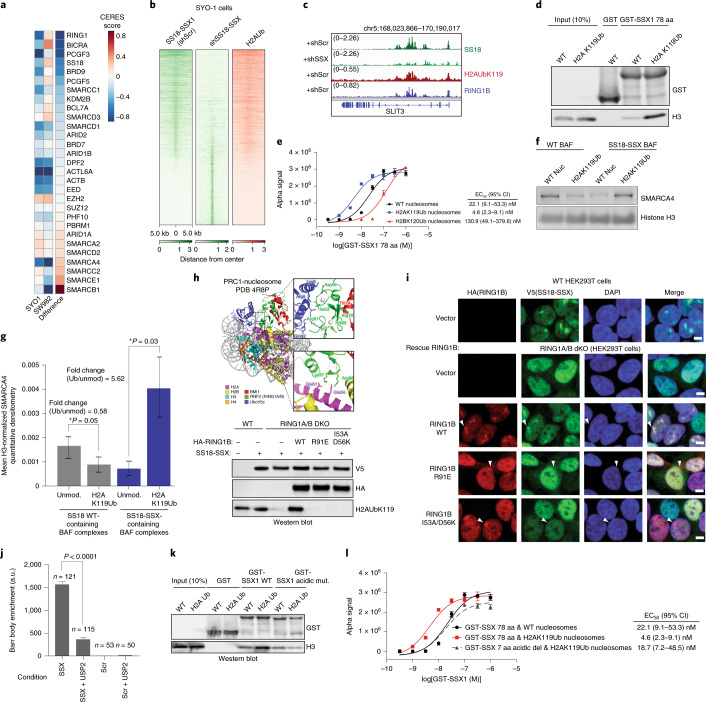
SSX preferentially binds H2AK119Ub-marked nucleosomes to promote BAF complex targeting to polycomb-repressed loci. **a**, CERES dependency scores from CRISPR-Cas9 fitness screens (Project Achilles). ‘Difference’ is the score calculated between SYO1 cells and SW982 cells. **b**, SS18 localization in SYO1 cells in control KD or shSS18-SSX conditions, aligned with H2AUb119 ChIP-seq. **c**, Example tracks at the *SLIT3* locus demonstrating co-localization of SS18-SSX BAF complexes, H2AK119Ub and RING1B. **d**, GST-SSX pull-down assays using either unmodified or H2AK119Ub nucleosomes. **e**, AlphaLISA assay performed with GST-SSX and 10 nM biotinylated unmodified, H2AK119Ub or H2BK120Ub nucleosomes. Error bars indicate mean ± s.d. of *n* = 4 experiments with half-maximum effective concentration (EC_50_) and 95% CI values calculated by nonlinear regression curve fitting. **f**, Pull-down assays using HA-SS18- or HA-SS18-SSX-bound BAF complexes incubated with either unmodified or H2AK119Ub-modified nucleosomes. SMARCA4 and H3 immunoblots are shown. **g**, Mean H3-normalized quantitative densitometry for SMARCA4 across SS18- and SS18-SSX-containing BAF complexes incubated with either unmodified or H2AK119Ub-modified nucleosomes. Error bars indicate mean ± s.d. of *n* = 5 independent experiments. *P* = 0.05 for SS18, unmodified versus H2AK119Ub; *P* = 0.03 for SS18-SSX, unmodified versus H2AK119Ub. **h**, Representation of PRC1 complex-nucleosome structure (PDB 4R8P), indicating mutated regions. Immunoblot of mutations that inhibit H2AK119Ub deposition. **i**, IF for RING1B (red) and V5-SS18-SSX (green), with DAPI nuclear stain in WT and RING1A-RING1B-dKO 293T cells with rescued conditions as indicated. Scale bars, 5 μm. **j**, Barr body localization for each condition: mean Barr body fluorescence intensity (a.u., arbitrary units) is plotted. Peptides were incubated after mock or USP2 treatment. Error bars indicate mean ± s.d of *n* = 121 and 115 cells examined for SSX and SSX + USP2 conditions, respectively. *P* < 0.0001 for SSX versus SSX + USP2). **k**, Pull-down experiments performed using either GST-SSX or GST-SSX acidic C-terminal 7 aa mutant with unmodified or H2AK119Ub nucleosomes, as indicated. **l**, AlphaLisa performed with GST-SSX or GST-SSXdel7aa and 10 nM unmodified or H2AK119Ub nucleosomes. Error bars represent mean ± s.d. of *n* = 4 experiments with EC_50_ and 95% CI values calculated by nonlinear regression curve fitting. Data points are shared with **e**. Uncropped gel images for **d**, **f**, **h** and **k** are provided in Supplementary Fig. 1. Raw binding data for **e**, **g**, **i** and **k** are available as Source data. Source data

**Extended Data Fig. 8:**
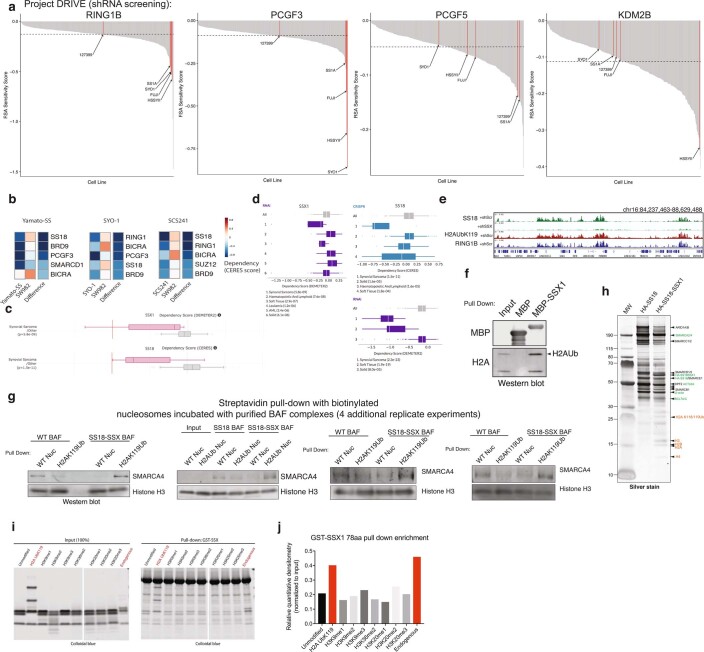
SS18-SSX-bound BAF complexes preferentially bind H2AK119Ub-marked nucleosomes. **a**, Waterfall dependency plots for RING1B, PCGF3, PCGF5 and KDM2B genes across n = 387 cell lines in Project DRIVE Dataset (Novartis). **b**, CERES dependency scores (fitness dropout) derived from genome-scale fitness screens performed using CRISPR-Cas9-based methods (Achilles, Broad Institute; https://depmap.org/portal/achilles/). Difference is the score calculated between SYO1, Yamato-SS, SCS241 (SS18-SSX + ) cells and SW982 cells (negative for fusion, histologic mimic). Blue, enriched for dependency. mSWI/SNF, PRC1, PRC2 members are shown. **c**, CERES and DEMETER Dependency scores for SSX1 and SS18 genes for CRISPR-Cas9 and RNAi datasets, respectively. Synovial sarcoma cell lines are indicated in pink; all other cell lines are represented in gray. **d**, CERES and DEMETER Dependency scores for SSX1 and SS18 genes for CRISPR-Cas9 and RNAi datasets, respectively. Synovial sarcoma and soft tissue (SS cell lines) exhibit preferential dependency. (Project DRIVE; https://oncologynibr.shinyapps.io/drive/). SS cell lines containing the SS18-SSX fusion oncoprotein are highlighted in red. **e**, H2AK119Ub and RING1B ChIP-seq tracks over selected loci, aligned with SS18 (BAF) localization in SYO1 cells treated with shScramble or shSS18-SSX. **f**, MBP-SSX1 (78aa) pull-down experiments indicate capture of histones, and specifically, H2AK119Ub species. **g**, Streptavidin-based pull-down experiments (n = 4 independent replicate experiments) using endogenous, fully-assembled HA-SS18- or HA-SS18-SSX-bound BAF complexes incubated with biotinylated WT nucleosomes (unmodified) or H2AK119Ub-modified nucleosomes. SMARCA4 and Histone H3 immunoblots are shown. **h**, Representative silver stain of the WT SS18 complexes and SS18-SSX fusion complexes isolated using ammonium sulfate nuclear extraction protocol. Identified proteins are labeled. **i**, Pull-down experiments performed using GST-SSX incubated with unmodified or a series of modified recombinant mononucleosomes, or endogenous mononucleosomes (mammalian, purified via MNase digestion from HEK-239T cells). **j**, Quantitative densitometry performed on experiment in Extended Data Fig. 8i. Uncropped gel images for all relevant panels are presented in Supplementary Figure 1.

Previously, we found that SS18-SSX-bound BAF complexes localize to polycomb-repressed regions^15^. The engagement between the conserved SSX basic region and the nucleosome acidic patch is not, in itself, sufficient to explain why SS18-SSX complexes are preferentially recruited to repressed chromatin. We therefore reasoned that the SSX-nucleosome acidic patch interaction might be augmented in some manner by the presence of specific histone repressive marks. To explore this possibility, we performed genome-scale CRISPR-Cas9-based screening and focused on genes encoding proteins that are responsible for decorating and maintaining repressive chromatin. These studies were performed in the SS cell line, SYO1, as well as in a cell line that is a SS histologic mimic lacking the SS18-SSX fusion, SW982 (Fig. 4a). Notably, we found that PRC1 subunits (specifically, RING1A and RING1B, as well as PCGF5 and PCGF3 components of PRC1.3 and PRC1.5 complexes) were selectively enriched as synthetic lethal dependencies in the SS cell line SYO1 as well as other SS cell lines including Yamato and SCS241 (Fig. 4a and Extended Data Fig. 8a,b). Importantly, all profiled SS cell lines exhibited significant dependency on SS18 and SSX (and hence the SS18-SSX fusion), relative to all other cell lines profiled (Extended Data Fig. 8c,d).

Given that the key histone modification placed by PRC1 is the H2AK119Ub mark, we sought to determine whether SSX exhibited any preferential binding to nucleosomes decorated with this modification. Notably, we found that in SS cell lines, H2AK119Ub directly co-localized with sites of SS18-SSX BAF complex occupancy (Fig. 4b,c and Extended Data Fig. 8e). This was consistent with our IF observations suggesting substantial co-localization at Barr bodies (Fig. 2c and Extended Data Figs. 3a,b,e, 4c and 6a). Indeed, pull-down experiments and AlphaLISA binding assays performed with GST-SSX 78 aa protein revealed higher affinity to H2AK119Ub-decorated nucleosomes relative to unmodified nucleosomes or H2BK120Ub nucleosomes (4.8-fold difference (Ub/unmod) by AlphaLISA) (Fig. 4d,e). Incubation of SSX 78 aa with mammalian mononucleosomes also captured the higher-molecular-weight H2AUb species (Extended Data Fig. 8f), as did SS18-SSX-bound BAF complexes (Fig. 1a,b and Supplementary Fig. 1). Importantly, endogenously purified SS18-SSX-bound BAF complexes enriched for binding of recombinant H2AK119Ub-modified nucleosomes over unmodified nucleosomes by 5.62-fold (*P* = 0.03) (Fig. 4f,g and Extended Data Fig. 8g,h), consistent with pull-down and fold changes detected by AlphaLISA binding studies performed with GST-SSX 78 aa protein above (Fig. 4d,e) and with the finding that SS18-SSX fusion target sites directly overlay H2AK119Ub sites genome-wide in SS cell lines (Fig. 4b). Finally, we performed a screen for SSX binding to a range of differentially marked recombinant mononucleosomes as well as mammalian (pooled) nucleosomes and again identified that GST-SSX 78 aa preferentially bound to H2AK119Ub and mammalian nucleosomes over unmodified nucleosomes or nucleosomes with other histone marks (Extended Data Fig. 8i–k).

**Extended Data Fig. 9:**
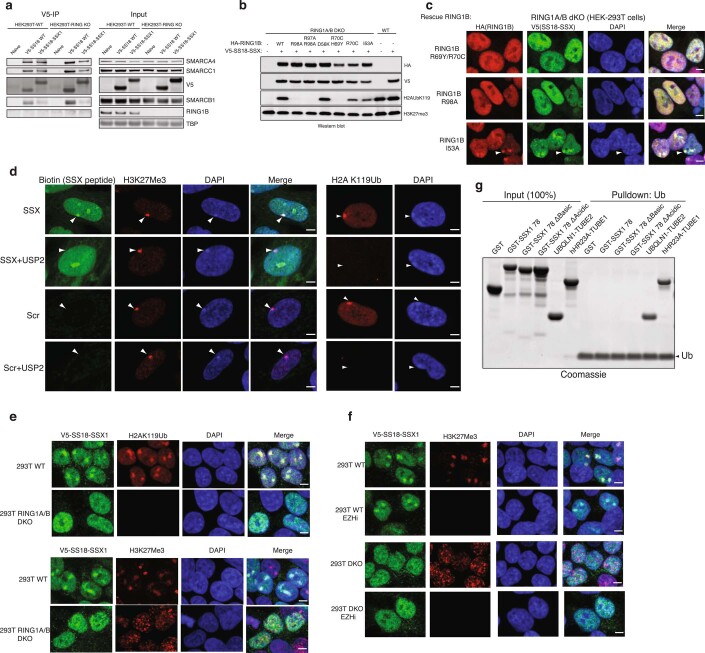
SSX targeting requires PRC1 complex-mediated H2AK119Ub placement. **a**, Immunoblots performed on V5 IP and Input protein levels in WT and RING1A and RING1B double KO (dKO) HEK293T cells. **b**, Immunoblot of representative, structurally-guided RING1B mutations which inhibit H2AK119Ub deposition partially, fully, or not at all. **c**, Immunofluorescence imaging for RING1B (red), V5-SS18-SSX (green), with DAPI nuclear stain, and merged images. **d**, Peptide hybridization experiments. Representative Images of SSX labeling of Barr bodies (inactive X) identified for each condition using H3K27me3 staining. Peptides (SSX or Scrambled) were incubated methanol-fixed cells, untreated or treated with USP2 deubiquitinating enzyme. **e**, V5-SS18-SSX, H2AK119Ub, and H3K27me3 IF studies performed in WT and RING1A and RING1B-dKO 293 T cells. **f**, DMSO control or EZH2 inhibitor treatment (to inhibit H3K27me3 placement) indicates no change to SS18-SSX foci localized to Barr bodies. **g**, Incubation of GST-SSX WT, SSX mutant variants, or UBQLN1-TUBE2 or hHR23A-TUBE1 (positive controls) with Ub-coated beads. Uncropped gel images for all relevant panels are presented in Supplementary Figure 1.

To understand the role of H2AK119Ub in SSX BAF localization, we next double-deleted the core, catalytic subunits of the PRC1 complex, RING1A and RING1B, using CRISPR-Cas9 in HEK293T cells (RING1A, RING1B-dKO HEK293T cells) and expressed SS18-SSX (Extended Data Fig. 9a). Following IF, we observed complete loss of SS18-SSX localization to Barr bodies as compared to RING1A,RING1B WT cells (Fig. 4g,h). To address whether the catalytic activity of PRC1 rather than PRC1 complex formation is required for SS18-SSX Barr body recruitment, we performed structure-guided mutagenesis to selectively disrupt the ubiquitin ligase activity of PRC1 and hence block its placement of H2AK119Ub (Fig. 4g). We designed a series of point mutations in RING1B to disrupt acidic patch recognition (R98A), zinc binding (H69Y, R70C) and the E2 binding interface (R91A and I53A/D56K^30^) (Fig. 4g,h and Extended Data Fig. 9b,c). Rescue of WT RING1B in RING1A-RING1B-dKO cells was able to completely rescue SSX localization. However, restoration of RING1B mutant variants affected SS18-SSX localization in a manner directly proportional to the degree to which these RING1B mutations impacted H2AK119Ub deposition. Significantly for this study, RING1A ligase-deficient R91E and I53A/D56K were able to form polycomb foci but were unable to recruit SS18-SSX, further highlighting the importance of the H2AK119Ub mark placement for SSX targeting. As controls, R98A and combined H69Y and R70C mutants had similar loss-of-function effects on SS18-SSX localization^31,32^ (Fig. 4g,h and Extended Data Fig. 9b,c). The widely used I53A mutant^33–35^ only partially attenuated H2A ubiquitination, and therefore had little effect on SSX targeting. As further support for a role for H2AK119Ub in SSX recruitment, we used a peptide hybridization assay performed on IMR90 cells pretreated with the deubiquitinating enzyme USP2. USP2-mediated removal of H2AK119Ub disrupted SSX peptide hybridization to Barr bodies specifically and without affecting its overall nuclear staining pattern, consistent with the general ability of SSX to bind unmodified nucleosomes via its acidic patch binding region. (Fig. 4i and Extended Data Fig. 9d). EZH2 inhibitor treatment performed in WT HEK293T or RING1A,RING1B-dKO HEK293T cells further highlighted the requirement for H2AKUb119 placement (and hence PRC1) rather than H3K27me3 and PRC2 activity (Extended Data Fig. 9e,f). Somewhat surprisingly, given the clear role for H2AK119Ub in recruiting SSX to chromatin, we did not observe direct binding between SSX and free ubiquitin, as assessed by a Ub-agarose pull-down assay (Extended Data Fig. 9g); however, it is conceivable that SSX only engages Ub in the context of H2AK119Ub nucleosomes, as seen with other chromatin readers such as Dot1L^36–38^), or that it might recognize specific features of the nucleosome core itself that are sterically or allosterically affected by the presence of the ubiquitylation mark.

Finally, given that disruption of the conserved C-terminal acidic region of SSX did not disrupt SSX-nucleosome binding (Fig. 2f), but did affect SS18-SSX-specific BAF complex targeting and resultant gene expression and proliferation (Fig. 2g–i) in a manner comparable to loss of the basic region (acidic patch binding region), we sought to determine whether this region mediates the preference of SSX for H2AK119Ub-decorated nucleosomes. Excitingly, we found that mutation of the C-terminal acidic region of SSX to alanines (that is DPEEDDE → AAAAAAA) relieved the preference of SSX for H2AK119Ub nucleosomes, while not altering general SSX binding to nucleosomes (Fig. 4j,k). These data collectively indicate that the conserved C-terminal acidic amino acids are required to drive the preference of SSX for H2AK119Ub nucleosomes and hence SS18-SSX-bound BAF complex targeting to repressive regions genome-wide, as observed in cells.

## Discussion

**Fig. 5:**
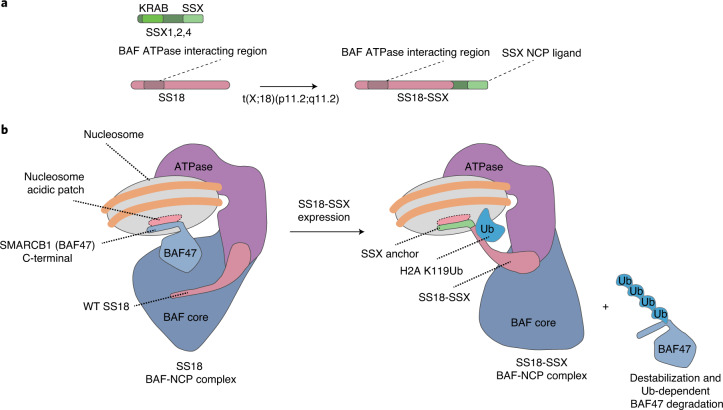
Model for SS18-SSX-bound BAF complex-nucleosome engagement. **a**, Schematic of SS18 WT and the SS18-SSX fusion oncoprotein. **b**, Model for BAF complex engagement on nucleosomes in WT and SS18-SSX fusion oncoprotein states. In WT complexes, the core module of BAF complexes engages the nucleosome acidic patch via the SMARCB1 C-terminal α-helical domain (aa 351–385). Upon expression of SS18-SSX, the SSX α-helical basic region (RLRERK) dominantly engages the acidic patch, displacing SMARCB1, which leads to its degradation, and changing the orientation of the BAF core module^23,28^ on the nucleosome. This SS18-SSX-specific conformation of BAF complexes exhibits strong preference for H2AUbK119-marked nucleosomes, conferring their preference for polycomb-decorated chromatin regions.

Here, we have identified an unexpected set of functionally critical properties of the fusion oncoprotein, SS18-SSX, the oncogenic driver of human SS (Fig. 5). We report an unusual case in which an additional nucleosome acidic patch binding domain is fused to a subunit of a major chromatin remodeling complex, the mammalian SWI/SNF (BAF) complex, conferring oncogenic properties to a tumor suppressor complex. Although several SNF2 helicase-based chromatin remodeling complexes are increasingly recognized to require the H2A-H2B nucleosome binding hub, we found that the minimal, conserved SSX 34 aa region dominantly binds the acidic patch of nucleosomes, and that SSX (or SS18-SSX-bound complexes) exhibit preferential binding to H2AK119Ub-modified nucleosomes, altering the nucleosome-SMARCB1 C-terminal α-helix interaction found in WT BAF complexes^7,27^ and resulting in higher-affinity nucleosome-binding properties augmented by specific repressive histone mark preferences^7^. These data, coupled with recent structural elucidation of yeast and human SWI/SNF complexes^28,39^, provide strong support for SS18-SSX-mediated displacement of SMARCB1 from the acidic patch and its destabilization at the nucleosome-proximal region of the core (base) module of BAF complexes. A high-resolution, three-dimensional structure of human BAF complexes containing SS18, as well as SS18-SSX, is required to define the full repertoire of structural changes to nucleosome-bound BAF complexes upon incorporation of SS18-SSX. This is particularly true given that the SS18 subunit is metazoan-specific and hence is not found in yeast complexes, nor has it been resolved in structural efforts to date.

The expression of full-length SSX (188 aa) is normally restricted to testes, where it plays a role in sperm development, potentially involving polycomb-driven XY-body repression through engagement of H2AK119Ub-decorated sex chromosomes^40^. Remarkably, this normal function of SSX as a binder of the nucleosome acidic patch and ‘reader’ of this repressive state is leveraged in SS to alter BAF chromatin remodeling complex localization and gene expression patterns. In testes, full-length SSX may normally function as a ligand for nucleosomes in this H2AK119Ub repressive state to promote further transcriptional repression through its N-terminal KRAB domain^41^. In the case of SS, the KRAB domain is replaced with essentially the whole ATPase module of the BAF chromatin remodeling complex via fusion to SS18. This unfortunate scenario leads to gain of repressive chromatin reading properties of BAF complexes, loss of normal BAF complex-nucleosome acidic patch engagement, tight affinity and longer residency times at normally polycomb-repressed regions, and the activation of genes found in these regions (Fig. 5).

We characterized the SSX 78 aa tail, particularly the conserved 34-aa C terminus (Fig. 2d), as a ligand of the nucleosome acidic patch that confers preferential affinity of SS18-SSX-bound complexes for H2AK119Ub-marked nucleosomes. Our data suggest two non-mutually exclusive explanations for this reading preference: (1) H2AK119Ub modification influences nucleosome structure by further exposing the acidic patch binding site or (2) SSX directly engages ubiquitin in the nucleosomal context. Although our present data indicate that SSX does not bind directly to free (bead-bound) ubiquitin (Extended Data Fig. 9g), this does not rule out the possibility of direct ubiquitin engagement by the acidic C-terminal region of SSX when SS18-SSX-bound complexes are docked on nucleosomes. In a similar manner, Dot1L will not bind free ubiquitin but will be poised to interact with H2BK120Ub during substrate engagement^36–38^. Determining the underlying basis of this binding preference requires structural characterization of SS18-SSX-bound human BAF complexes or at least high-resolution structures of SS18-SSX on H2AK119Ub-marked nucleosomes. Nonetheless, the present results suggest that SSX acts as a binding ligand for the acidic patch on H2AK119Ub-decorated nucleosomes and that this property underlies the chromatin localization, gene expression and synthetic lethal profiles of this tumor type.

The biochemical and structural properties of the SSX fusion partner elucidated here underpin the dependency of SS on PRC1 complex activity that has been detected in fitness screening efforts and in our own structure-guided mutagenesis studies. In contrast to other reports^22^, we do not find direct binding to PRC1 by the SS18-SSX fusion (or by SSX specifically), nor a selective dependency on the PRC1 subunit KDM2B; rather, we find that SS18-SSX-bound complexes bind preferentially and tightly to H2AK119Ub-marked nucleosomes and hence require PRC1 complex-mediated placement of the H2AUbK119 mark. Furthermore, we do not detect peptides corresponding to PRC1 or PRC2 in MS experiments examining SS18-SSX binding partners; instead, these data reveal highly abundant peptides corresponding to histones and ubiquitin itself, along with enrichment of peptides corresponding to the ATPase module subunits of BAF complexes (SMARCA4, BCL7A, β-actin, ACTL6A) to which SS18 is tethered. The increased abundance and high affinity of SS18-SSX-containing BAF complexes over PRC1-decorated sites, and hence the frequency of molecules co-localized on chromatin, may help reconcile these previous suggestions.

In summary, we have identified an unanticipated nucleosome acidic patch-binding function of SSX, an oncoprotein fusion partner lacking canonical DNA-binding or chromatin-reader domains, that alters the function of BAF chromatin remodeling complexes and activates oncogenic programs in a cancer-specific manner. Our results suggest that inhibition of the SSX- or SS18-SSX-bound BAF complex-H2AK119Ub nucleosome interactions using small molecules or peptides may prove to be a viable therapeutic strategy for SS.

## Methods

### Cell lines and cell culture

The two SS cell lines, Aska and SYO1, were generous gifts from K. Itoh, N. Naka and S. Takenaka (Osaka University, Japan) and A. Kawai (National Cancer Center Hospital, Japan), respectively. The CRL7250 human fibroblast cell line was obtained from B. Gryder and J. Khan (National Cancer Institute, Bethesda, MD). The HEK293T cell line was purchased ATCC (CRL-3216). Each cell line was cultured using standard protocols in DMEM medium (Gibco) supplemented with 10–20% FBS, 1% Glutamax (Gibco), 1% sodium pyruvate (Gibco) and 1% penicillin-streptomycin (Gibco) and grown in a humidified incubator at 37 °C with 5% CO_2_. All cell lines were routinely tested for mycoplasma contamination, confirmed to be negative before all experiments, and were authenticated before use.

### Stable gene expression and shRNA KD constructs

Constitutive expression of SS18 WT (*SS18*), SS18-SSX1 (*SS18-SSX1*) and SS18-SSX1 mutations with HA or V5 N-terminus tag was obtained using an EF1alpha-driven expression vector (modified from Clonetech, dual Promoter EF-1a-MCS-PGK-Puro or EF-1a-MCS-PGK-Blast) expressed in cells by lentiviral infection and selected with puromycin (2 μg ml^−1^) or blasticidin (10 µg ml^−1^). Constitutive expression of shRNA hairpins targeting the 3′UTR region of SSX of the SS18-SSX fusion (5′-CAGTCACTGACAGTTAATAAA-3′) or a scramble non-targeting control (5′-CCTAAGGTTAAGTCGCCCTCGCTCGAGCGAGGGCGACTTAACCTTAGG-3′) was obtained using lentiviral infection of the pLKO.1 vector with puromycin (2 μg ml^−1^) selection.

### Lentivirus generation and harvesting

Lentivirus production was obtained from PEI (Polysciences) transfection of HEK293T LentiX cells (Clontech) with co-transfection of the packaging vectors pspax2 and pMD2.G along with the gene delivery vector. Viral supernatants were collected 72 h after transfection, underwent ultracentrifugation at 20,000 r.p.m. for 2.5 h at 4 °C to concentrate, and then virus pellets were resuspended in PBS. For infection, the viral pellets were added to cells in a dropwise manner in the presence of polybrene (10 μg ml^−1^). After 48 h, medium containing the lentivirus was replaced and infected cells were selected by addition of puromycin (2 μg ml^−1^) or blasticidin (10 µg ml^−1^).

### Western blot analysis

Detection of proteins by western blot analysis was achieved using standard protocols with primary antibodies (Supplementary Table 4). Samples were separated on 4–12% Bis-Tris SDS–PAGE gel (Invitrogen) and transferred to polyvinylidene difluoride membrane. The membranes were then blocked in 5% milk and incubated with primary antibody in PBST overnight at 4 °C. Following incubation with the primary antibody, membranes were washed three times in PBST, incubated with IRDye (LI-COR Biosciences) secondary antibodies for 3 h, washed three times in PBST with a final PBS wash, and then visualized by the LI-COR Odyssey Imaging System (LI-COR Biosciences).

### Cell lysate collection

Whole-cell extractions were obtained by washing harvested cell pellets with PBS pH 7.4, resuspending them in whole-cell lysis buffer (PBS pH 7.4 and 1% SDS) and then heating for 3 min at 95 °C. Lysates were sonicated until fully liquid. NEs were obtained by suspending the harvested cells in buffer 0 (50 mM Tris pH 7.5, 0.1% NP40, 1 mM EDTA, 1 mM MgCl_2_ with protease inhibitor cocktail, 1 mM DTT and 1 mM phenylmethylsulfonyl fluoride (PMSF)), centrifuging at 5,000 r.p.m. for 5 min at 4 °C and discarding the supernatant. The pellets (nuclei) were resuspended in EB300 buffer (50 mM Tris pH 7.5, 0.1% NP40, 1 mM EDTA, 1 mM MgCl_2_, 300 mM NaCl with protease inhibitor cocktail, 1 mM DTT and 1 mM PMSF), vortexed, incubated on ice, centrifuged at 15,000 r.p.m. for 10 min at 4 °C, then supernatant containing the NE was collected.

### Nuclear extraction

NEs for 293T V5-SS18 WT and V5-SS18-SSX1 cells were prepared as previously described^15^. Specifically, cells were scraped from plates, washed with cold PBS, pelleted at 3,000 r.p.m. for 5 min at 4 °C and resuspended in buffer A hypotonic buffer (50 mM HEPES, pH 7.6, 25 mM KCl, 10% glycerol, 0.1% NP40, 0.05 mM EDTA, 5 mM MgCl_2_ supplemented with protease inhibitor cocktail, and 1 mM PMSF). Lysates were pelleted at 3,000 r.p.m. for 5 min at 4 °C. Supernatants were discarded and nuclei were resuspended in buffer C high-salt buffer (10 mM HEPES, pH 7.6, 100 mM KCl, 10% glycerol, 0.5 mM EDTA, 3 mM MgCl_2_ supplemented with protease inhibitor and 1 mM PMSF). Lysates were incubated at 4 °C at constant rotation. Lysates were then pelleted at 40,000 r.p.m. for 1 h at 4 °C. Supernatants were collected and mixed with (NH_4_)_2_SO_4_ at 300 mg ml^−1^ for 30 min. Samples were pelleted at 15,000 r.p.m. for 30 min and supernatant was discarded. Protein concentrations were quantified via bicinchonic acid (BCA) assay (Pierce). Finally, samples were supplemented with 1 mM DTT.

### Co-immunoprecipitations

Nuclear extracts were quantified by Bradford assay and 150–200 μg of protein was incubated with 2 μg of antibody in EB300 buffer (50 mM Tris pH 7.5, 0.1% NP40, 1 mM EDTA, 1 mM MgCl_2_, 150 mM NaCl with protease inhibitor cocktail, 1 mM DTT and 1 mM PMSF) overnight at 4 °C. Each sample was then incubated with Protein G Dynabeads (Thermo Scientific) for 2–3 h. Beads were washed three times with EB300 buffer followed by elution with 20 μl of elution buffer (NuPage LDS buffer (2×) (Life Technologies) containing 100 mM DTT and water).

### Cell proliferation assay

To measure cell proliferation following lentiviral infection, 2.5 × 10^4^ cells per well were seeded in 12-well plates following 48-h exposure to lentivirus and 5-day selection with puromycin or blasticidin, with day 7 denoting the day cells were plated after infection and selection. The cell viability in three wells was then measured using a Vi-CELL Cell Counter (Beckman) every 72 h.

### Differential salt extraction

Following collection of 5.0 × 10^7^ cells, cells were resuspended in elution 0 buffer (50 mM Tris-HCl pH 7.5, 1 mM EDTA, 0.1% NP40 with protease inhibitor cocktail and 1 mM PMSF), incubated on ice for 5 min and pelleted by centrifugation. The supernatant was collected (0 mM fraction) and the cell pellet was resuspended in elution 150 buffer (50 mM Tris-HCl pH 7.5, 150 mM NaCl, 1 mM EDTA, 0.1% NP40 with protease inhibitor cocktail and 1 mM PMSF) and vortexed. This process was repeated sequentially with elution 300 buffer, elution 500 buffer and elution 1,000 buffer that contained increasing concentrations of NaCl to obtain 0, 150, 300, 500 and 1,000 mM NaCl soluble fractions. Each of these soluble fractions, along with a total sample (5 × 10^6^ cells in elution buffer) and the chromatin pellet (non-soluble material remaining following extraction with 1,000 mM NaCl) fractions, was denatured in SDS to a final concentration of 1%, then the protein was quantified by a Pierce BCA Protein Assay Kit (Thermo Fisher Scientific) and analyzed (1.5 μg of protein) by immunoblot.

### Purification of mSWI/SNF (BAF) complexes

Stable HEK293T cell lines expressing, by lentiviral infection, HA-SS18 WT or HA-SS18-SSX1 were grown in 150-mm dishes. Complexes were purified using methods previously described with a few modifications^23^. Confluent plates were scraped to remove cells and cells were washed with PBS. The cell suspension was spun down by centrifugation at 3,000 r.p.m. for 5 min at 4 °C and pellets were resuspended in hypotonic buffer (10 mM Tris-HCl pH 7.5, 10 mM KCl, 1.5 mM MgCl_2_, 1 mM DTT and 1 mM PMSF) and incubated on ice for 5 min. Following incubation, the cell suspension was spun down by centrifugation at 5,000 r.p.m. for 5 min at 4 °C, and pellets were resuspended in 5× volume of fresh hypotonic buffer (with protease inhibitor cocktail), then cells were homogenized using a Dounce homogenizer (glass). Cell suspension was layered onto hypotonic buffer sucrose cushion made with 30% sucrose (wt/vol), spun down by centrifugation at 5,000 r.p.m. for 1 h at 4 °C, followed by removal of the cytosol-containing layer. The nuclei-containing pellets were resuspended in high-salt buffer (50 mM Tris-HCl pH 7.5, 300 mM KCl, 1 mM MgCl_2_, 1 mM EDTA, 1 mM 1% NP40, 1 mM DTT, 1 mM PMSF and protease inhibitor cocktail) and then the homogenate was rotated for 1 h at 4 °C. Homogenates were then spun down by centrifugation at 20,000 r.p.m. for 1 h at 4 °C in an SW32Ti rotor (Beckman Coulter). The soluble proteins, consisting of the NE fraction, were separated from the insoluble chromatin pellet, consisting of the CHR fraction. The chromatin pellet was further solubilized by treatment with benzonase (Sigma Aldrich) for 30 min and subsequently additional KCl was added to a final concentration of 700 mM (50 mM Tris-HCl pH 7.5, 700 mM KCl, 1 mM MgCl_2_, 1 mM EDTA, 1 mM, 1% NP40, 1 mM DTT, 1 mM PMSF and protease inhibitor cocktail), and sonicated three times for 30 s with 5 min intervals. The solubilized chromatin fraction was then spun down by centrifugation at 20,000 r.p.m. for 1 h at 4 °C in an SW32Ti rotor (Beckman Coulter) and the supernatant collected. The collected NE and chromatin fractions were filtered with a 0.45-μm filter and rotated overnight at 4 °C with HA magnetic resin. HA beads were washed in high-salt buffer and eluted with 1 mg ml^−1^ of HA peptide four times for durations of 1.5 h each. Eluted proteins were then subjected to density gradient centrifugation or dialysis.

### Colloidal Blue and silver stain

HA-SS18 WT and HA-SS18-SSX1 mSWI/SNF complexes were purified via HA-epitope-dependent complex purification. Importantly, for Fig. 1a, the same number of cells were used for both HA-SS18 WT and HA-SS18-SSX expressing cells, and nuclear material from both cell lines was split into NE and CHR fractions, representing an equal total amount of complexes in the nucleus. Hence, equal input/output loading by volume was achieved. Samples were run on a 4–12% Bis-Tris SDS–PAGE gel, stained using a Colloidal Blue kit or SilverQuest Silver Staining Kit (Invitrogen), and imaged using a LI-COR Odyssey Imaging System (LI-COR Biosciences) or an Epson-Perfection V600 Photo scanner, respectively.

### Density sedimentation gradients

Purified protein complexes were added to the top of a linear, 11 ml 10–30% glycerol gradient containing 25 mM HEPES pH 7.9, 0.1 mM EDTA, 12.5 mM MgCl_2_, 100 mM KCl with 1 mM DTT and protease inhibitors. Gradient tubes were placed into an SW41 rotor (Beckman Coulter) and spun by centrifugation at 40,000 r.p.m. for 16 h at 4 °C. Fractions of 550 µl volume were collected sequentially from the top of the gradient, then 100 µl of each fraction was concentrated with 10 µl of Strataclean beads (Agilent Technologies, 400714), loaded and run on an SDS–PAGE gel, then analyzed by SYPRO Ruby Protein Gel Stain (Thermo Fisher Scientific) and scanned using a Typhoon FLA 9500 scanner.

### Mass spectrometry proteomics analysis of purified complexes

Equal amounts of purified HA-SS18 WT and HA-SS18-SSX1 complexes were loaded onto SDS–PAGE gels from both the NE and CHR fractions. Samples were migrated into the gel for a length of 2 cm, gels were stained with Colloidal Blue stain and protein bands were excised for protein detection by MS. The samples were then prepared and the data analyzed by the Taplin Biological Mass Spectrometry Facility (directed by S. Gygi; Harvard Medical School).

### Protein and peptide pull-downs

Recombinant purified proteins with affinity tags (MBP or GST) or biotinylated peptides were purified using magnetic beads (maltose, glutathione or streptavidin, respectively) by incubation in EB150 buffer (50 mM Tris-HCl pH 7.5, 150 mM NaCl) 1 mM EDTA, 0.1% NP40 with protease inhibitor cocktail and 1 mM PMSF at 4 °C overnight. The flow-through was removed, the immobilized bait was incubated with 1–2 µg of purified mammalian mononucleosomes from HEK293T cells, recombinant mononucleosomes (EpiCypher, 16-0006), recombinant H2AK119Ub mononucleosomes (EpiCypher, 16-0020) or recombinant protein for 3 h at 4 °C, and the beads were washed three times with EB150 buffer and then eluted in 2× LDS with 200 mM DTT with heating at 95 °C for 5 min. The pull-downs were then visualized by immunoblot analysis or Colloidal Blue staining.

### Peptide competition experiments

The peptide competition experiments were set up in a similar manner as the peptide pull-down experiments, with the following exceptions: SSX1 (aa 55–78) or SMARCB1-CC (aa 351–385) biotin-labeled peptides at 10 μM in EB150 were bound to streptavidin Dynabeads (Pierce streptavidin magnetic beads, Thermo Scientific) in parallel to 1–2 μg of mononucleosomes incubated with LANA, SSX (aa 155–188) or SMARCB1-CC (aa 351–385) peptide (KE Biochem) at varying concentrations ranging from 0 to 30 μM overnight at 4 °C. Beads were washed three times in EB150 medium and resuspended with the mononucleosomes-LANA peptide solutions. The suspension was rotated for 3–5 h at 4 °C. The beads were washed five times in EB150 medium, and eluted in sample buffer (2× LDS with 200 mM DTT) to load onto 10–20% tricine gels.

### Quantitative targeted mass spectrometry

Mammalian mononucleosomes purified from MBP-SSX1 78aa pull-downs along with representative input samples were prepared and analyzed by the targeted MS pipeline described previously^42^. Briefly, samples were prepared by histone extraction by acid precipitation followed by protein digestion from incubation with trypsin. To these prepared samples, synthesized isotopically labeled peptides of histone tails with numerous modifications were added at a known quantity. Each sample was then separated using a Proxeon EASY-nLC 1000 UHPLC system (Thermo Scientific) and detected with a Q Exactive mass spectrometer (Thermo Scientific). The fold change in abundance of each histone peptide from the input sample compared to the pull-down was calculated from the light:heavy ratio in detected peak size.

### Detection of nucleosome acidic patch interactions by photocrosslinking

Details of the design and preparation of diazirine-containing nucleosomes for photocrosslinking studies is described elsewhere^6^. Briefly, diazirine-containing recombinant nucleosomes (0.5 μM) were incubated with biotinylated SSX peptides (12.5 μM) in binding buffer (20 mM HEPES, pH 7.9, 4 mM Tris, pH 7.5, 150 mM KCl, 10 mM MgCl_2_, 10% glycerol and 0.02% (vol/vol) IGEPAL CA-630) at 30 °C for 30 min and cooled on ice for 5 min. The reaction mixtures were then irradiated at 365 nm for 10 min. Reactions were analyzed by western blotting employing IRDye 800CW streptavidin on a LI-COR Odyssey Infrared Imager. Additional details are provided in ref. ^6^.

### Immunofluorescence

The IF images were obtained as described previously^43^. Following lentiviral infection and/or drug treatment, cells were prepared by fixation in 3% PFA-PBS and then permeabilized with PBS 0.1% NP40. Following incubation with primary antibodies, anti-rabbit Alexa Fluor 594 and anti-mouse Alexa Fluor 488 (Life Technologies) secondary antibodies were used for visualization. Staining with 4′,6-diamidino-2-phenylindole (DAPI) was used to visualize nuclei. Images were acquired using a Zeiss Axio Imager Z2 microscope and images were processed using the ImageJ program (NIH).

### Fluorescent recovery after photobleaching

FRAP experiments were carried out in the same manner as described previously^44^. Briefly, HEK293T cells expressing GFP-SS18 WT or GFP-SS18-SSX1 by lentiviral infection or Aska cells co-expressing BRG1-Halo fusion with pLKO.1 shScramble control or shSSX were imaged to measure the mean FI of a defined nuclear region pre- and post-photobleaching at 5-s intervals. The relative fluorescence intensity (RFI) for each image was calculated by normalizing the maximal difference in FI post-bleaching to 1. The *t*_1/2_ values and mobile fractions were determined using the software Prism (GraphPad Software) from *n* = 27–30 cells in each condition over two biological replicates.

### Chromatin immunoprecipitation

For chromatin immunoprecipitation (ChIP) experiments, prepared cells were collected following 48 h of lentiviral infection and 5-d selection (unless otherwise indicate) with puromycin or blasticidin. Capture of chromatin-bound proteins was performed using standard protocols (Millipore). Briefly, cells were crosslinked with 1% formaldehyde for 10 min at 37 °C, the reaction was quenched by addition of 125 mM glycine for 5 min and then five (for synovial sarcoma cell lines) or ten (for fibroblast cell lines) million fixed cells were used per experiment. Chromatin was fragmented by sonication with a Covaris E220 system and the solubilized chromatin was incubated with a primary antibody overnight at 4 °C to form antibody–chromatin complexes. These complexes were incubated with protein G Dynabeads (Thermo Scientific) for 3 h at 4 °C then the beads were washed three times and eluted. The samples then underwent crosslink reversal, treatment with RNase A (Roche) and treatment with proteinase K (Thermo Scientific) followed by DNA capture with AMP Pure beads (Beckman Coulter).

### RNA isolation from cell lines

Cells (1 × 10^6^) were collected following 48 h of lentiviral infection and five days (seven days post-infection) of selection with puromycin or blasticidin for extraction of RNA for RNA-seq experiments. Samples for RNA-seq were prepared in biological duplicate (collected using independent production of lentivirus, infection, selection and cell culture). Total RNA was collected using the RNeasy Mini Kit (Qiagen) following homogenization of cell lysates using the QIAshredder (Qiagen).

### Library preparation and sequencing for RNA and ChIP samples

Library preparations for next-generation sequencing of RNA-seq samples were performed using an NEBNext poly(A) mRNA magnetic isolation module (New England BioLabs) to purify mRNA from 1 µg of total RNA isolated from cells. This isolated mRNA was then used with the NEBNext Ultra II Directional RNA Library prep kit for Illumina (New England BioLabs) to generate DNA. The DNA from these prepared RNA samples as well as the ChIP-seq samples were then prepared for sequencing using the NEBNext Ultra II (New England BioLabs) to amplify and barcode each sample. The fragments sizes were determined using a D1000 ScreenTape system (Agilent) and the DNA quantified by Kapa Library Quantification Kit Illumina platforms (Kapa Biosystems). The samples were then diluted and loaded on a buffer cartridge for 75-bp single-end sequencing on the NextSeq 500 system (Illumina).

### CRISPR-Cas9 and shRNA synthetic lethal screening data analyses

CRISPR-Cas9 datasets (Avana-19Q3) were obtained from the Project Achilles data portal (https://depmap.org/portal/achilles/). Fitness (CERES) scores were extracted for each cell line and hierarchical clustering was performed using complete linkage and correlation as a distance measure. Heatmaps were generated using pheatmap in RStudio. DRIVE data are publicly available and can be downloaded from the Novartis DRIVE data portal (https://oncologynibr.shinyapps.io/drive/). Waterfall plots were generated using ggplot2 in RStudio.

### Purification of mammalian mononucleosomes

Mammalian mononucleosomes were purified from HEK293T cells transfected with pCDNA3 Flag-H2A as previously described^45^. Cells were scraped from plates, washed with cold PBS, and centrifuged at 5,000 r.p.m. for 5 min at 4 °C. Pellets were resuspended in hypotonic buffer (EB0: 50 mM Tris-HCl, pH 7.5, 1 mM EDTA, 1 mM MgCl_2_, 0.1% NP40 supplemented with 1 mM DTT, 1 mM PMSF and protease inhibitor cocktail) and incubated for 5 min on ice. The suspension was centrifuged at 5,000 r.p.m. for 5 min at 4 °C, and pellets were resuspended in five volumes of high-salt buffer (EB420: 50 mM Tris-HCl, pH 7.5, 420 mM NaCl, 1 mM MgCl_2_, 0.1% NP40 with supplemented with 1 mM DTT and 1 mM PMSF containing protease inhibitor cocktail). The homogenate was incubated on a rotator for 1 h at 4 °C. The supernatant was centrifuged at 20,000 r.p.m. (30,000*g*) for 1 h at 4 °C using an SW32Ti rotor, then the supernatant was discarded and the chromatin pellet washed in MNAse buffer (20 mM Tris-HCl pH 7.5, 100 mM KCl, 2 mM MgCl_2_, 1 mM CaCl_2_, 0.3 M sucrose, 0.1% NP40 and protease inhibitor cocktail) three times. Following MNase treatment (3 U ml^−1^ for 30 min at room temperature (RT), Sigma Aldrich), the reaction was quenched with 5 mM of EGTA and 5 mM of EDTA. The samples were then centrifuged at 20,000*g* for 1 h at 4 °C to obtain the soluble chromatin fraction. This fraction was then incubated with M2 anti-Flag magnetic beads (Sigma) overnight. Beads were washed with EB300 medium (50 mM Tris-HCl, pH 7.5, 300 mM NaCl, 1 mM MgCl_2_, 0.1% NP40 supplemented with 1 mM DTT and 1 mM PMSF containing protease inhibitor cocktail) and eluted with EB300 containing 0.2 mg ml^−1^ of Flag peptide three times for 1.5 h at 4 °C. Elution fractions were loaded onto a 10–30% glycerol gradient^23^ and fractions containing mononucleosomes were isolated and concentrated using ultra concentrators (Amicon, EMD Millipore).

### Restriction enzyme accessibility assay nucleosome remodeling assay

SMARCA4 (BRG1) levels of the ammonium sulfate nuclear extracts were normalized via BCA protein quantification and silver stain analyses for HA-SS18 and HA-SS18-SSX conditions. Protein was diluted to a final reaction concentration of 150 μg ml^−1^ in REAA buffer (20 mM HEPES, pH 8.0, 50 mM KCl, 5 mM MgCl_2_) containing 0.1 mg ml^−1^ BSA, 1 mM DTT, 20 nM nucleosomes (EpiDyne nucleosome remodeling assay substrate ST601-GATC1, EpiCypher). The REAA mixture was incubated at 37 °C for 10 min, and the reaction was initiated using 1–2 mM ATP (Ultrapure ATP, Promega) and 0.005 U ml^−1^ DpnII restriction enzyme (New England Biolabs). The REAA reaction mixture was quenched with 20–24 mM EDTA and placed on ice. Proteinase K (Ambion) was added at 100 mg ml^−1^ for 30–60 min, followed by either AMPure bead DNA purification and D1000 HS DNA ScreenTape analysis (Agilent) or mixing with GelPilot Loading Dye (QIAGEN) and loading onto 8% TBE gel (Novex 8%TBE Gels, Thermo Fisher). TBE gels were stained with either SYBR-Safe (Invitrogen) or Syto-60 red fluorescent nucleic acid stain (Invitrogen), followed by imaging with ultraviolet light on an Alpha Innotech AlphaImager 2200 and/or with 652-nm light excitation on a Li-Cor Odyssey CLx imaging system (LI-COR).

### ATPase assays

ATPase consumption assays were performed using the ADP-Glo kinase assay kit (Promega) according to the manufacturer’s instructions. We used the same conditions as for the REAA nucleosome remodeling assay described above. Following incubation with desired substrates for 40 min at 37 °C, a 1× volume of ADP-Glo reagent was used to quench the reaction and incubated at RT for 40 min. A 2× volume of the kinase detection reagent was then added and incubated at RT for 1 h. Luminescence readout was recorded. The substrates used for this assay measuring nucleosome-bound ATPase activity were purified recombinant mononucleosome (EpiDyne nucleosome remodeling assay substrate ST601-GATC1, EpiCypher, cat. no. 16-4101). NE material was used at 150 μg for each ARID1A immunoprecipitation (IP) using ARID1A antibody (Cell Signaling, cat. no. 12354S).

### Preparation of peptides

Custom peptide sequences were prepared using standard synthesis techniques from KE Biochem. The peptides were confirmed to have >95% purity by HPLC and obtained as a white to off-white lyophilized powder. The powder was resuspended in DMSO (Sigma) for use in experiments.

### Expression and purification of recombinant proteins

DNA constructs of human SSX1 aa 111–188 and related mutates in pGEX-6P2 expression vector were transformed in *Escherichia coli* BL21 (DE3) cells and overexpressed in TB medium in the presence of 100 μg ml^−1^ of ampicillin. Cells were grown at 37 °C to an optical density at 600 nm of 0.6, cooled to 17 °C, induced with 500 μM IPTG, incubated overnight at 17 °C, collected by centrifugation and stored at −80 °C. Cell pellets were resuspended in buffer A (25 mM HEPES, pH 7.5, 200 mM NaCl, 5% glycerol and 0.5 mM TCEP) supplemented with 1 mM PMSF, lysed in a Microfluidizer (Microfluidics) and centrifuged at 16,000*g* for 45 min. Glutathione sepharose beads (GE Healthcare) were incubated with lysate supernatant for 90 min to captured GST-tagged proteins and washed with buffer A. Beads with bound protein were transferred to an FPLC-compatible column and the bound protein was washed with high-salt buffer (buffer A containing 1 M NaCl) followed by elution with buffer A supplemented with 15 mM glutathione (Sigma). Eluted protein fractions were collected, concentrated and purified by size exclusion chromatography using a Superdex 75 10/300 column (GE Healthcare) equilibrated with buffer A. Eluted protein was incubated with GST-3C protease at 4 °C overnight. Cleaved samples were incubated with a second round of glutathione beads to remove GST-3C and free GST, and the desired protein product contained within the flow-through fractions was further purified by ion-exchange chromatography using a mono-Q column (GE Healthcare). Fractions containing the cleaved protein product were pooled, concentrated and stored at −80 °C.

### Peptide hybridization assay

IMR90 fibroblast were grown on coverslips, washed with PBS and fixed using 100% ice-cold methanol for 3 min. Coverslips were then washed with IF wash buffer (PBS 0.1% NP40 1 mM sodium azide) three times. Selected groups were treated with 200 ng ml^−1^ of recombinant USP2 catalytic domain (Boston Biochem) for 1 h. Coverslips were then washed three times with IF wash buffer and incubated with 2 μM of biotinylated peptides. Coverslips were subsequently washed three times with IF wash buffer and fixed in 3% PFA-PBS for 20 min. The rest of the procedure followed according to standard IF protocol. In brief, following incubation with primary antibodies, anti-rabbit Alexa Fluor 594 and streptavidin Alexa Fluor 488 (Life Technologies) secondary antibodies/reagent were used for visualization of primary antibodies or biotinylated peptides. Staining with DAPI was used to visualize nuclei. Images were acquired using a Zeiss Axio Imager Z2 microscope and images were processed using the ImageJ program (NIH).

### Statistics

All graphical representations of data and statistical analyses were performed using either Mann–Whitney U-tests or two-tailed Student’s *t*-test. Error bars representing standard deviations, the number of events, number of biological replicates and *P* values are indicated in the figure legends.

### Reporting Summary

Further information on research design is available in the Nature Research Reporting Summary linked to this article.

## Online content

Any methods, additional references, Nature Research reporting summaries, source data, extended data, supplementary information, acknowledgements, peer review information; details of author contributions and competing interests; and statements of data and code availability are available at 10.1038/s41594-020-0466-9.

## Supplementary information

Supplementary Information Supplementary Fig. 1 and Note.

Reporting Summary

Supplementary Table 1 Purification of HA-SS18-containing or HA-SS18-SSX-containing complexes in nuclear extract (NE) or chromatin-bound (CHR) fractions.

Supplementary Table 2 Raw data for quantitative histone mass-spectrometry analyses performed on MBP-SSX 78 aa protein relative to MBP control protein incubated with mammalian mononucleosomes.

Supplementary Table 3 Proteomic IP-mass spectrometry for BRG1 IPs in human fibroblasts and Aska-SS cells.

Supplementary Table 4 Detailed information for all antibodies used in this study.

## Data Availability

All sequencing data have been deposited with the Gene Expression Omnibus under accession no. GSE139055. Proteomic data have been deposited in PRIDE with accession no. PXD018715. Uncropped gel and blot images are presented in Supplementary Fig. 1. Source data are provided with this paper.

## Source data

Source Data Fig. 1 Statistical source data Source Data Fig. 2 Statistical source data Source Data Fig. 3 Statistical source data Source Data Fig. 4 Statistical source data Source Data Extended Data Fig. 1 Statistical source data Source Data Extended Data Fig. 2 Statistical source data Source Data Extended Data Fig. 4 Statistical source data Source Data Extended Data Fig. 6 Statistical source data Source Data Extended Data Fig. 7 Statistical source data
