# Author Correction: The nucleosome acidic patch and H2A ubiquitination underlie mSWI/SNF recruitment in synovial sarcoma

**DOI:** 10.1038/s41594-020-00540-y

**Published:** 2020-12-03

**Authors:** Matthew J. McBride, Nazar Mashtalir, Evan B. Winter, Hai T. Dao, Martin Filipovski, Andrew R. D’Avino, Hyuk-Soo Seo, Neil T. Umbreit, Roodolph St. Pierre, Alfredo M. Valencia, Kristin Qian, Hayley J. Zullow, Jacob D. Jaffe, Sirano Dhe-Paganon, Tom W. Muir, Cigall Kadoch

**Affiliations:** 1 Department of Pediatric Oncology, Dana-Farber Cancer Institute and Harvard Medical School, Boston, MA USA; 2 Broad Institute of MIT and Harvard, Cambridge, MA USA; 3 Program in Chemical Biology, Harvard University, Cambridge, MA USA; 4 Department of Chemistry, Princeton University, Princeton, NJ USA; 5 Department of Cancer Biology, Dana-Farber Cancer Institute, Boston, MA USA; 6 Department of Cell Biology, Harvard Medical School, Boston, MA USA; 7 Biological and Biomedical Sciences Program, Harvard Medical School, Boston, MA USA

**Keywords:** Chromatin remodelling, Cancer, Transcription

Correction to: *Nature Structural & Molecular Biology* 10.1038/s41594-020-0466-9, published online 3 August 2020.

This paper was originally published under standard Springer Nature copyright (© The Author(s), under exclusive licence to Springer Nature America, Inc.). It is now available as an open-access paper under a Creative Commons Attribution 4.0 International license, © The Author(s). The error has been corrected in the print, HTML and PDF versions of the article.

