# Supplementary material for: The nucleosome acidic patch and H2A ubiquitination underlie mSWI/SNF recruitment in synovial sarcoma
Source: Nat Struct Mol Biol. 2020 Aug 3;27(9):836–45. doi: 10.1038/s41594-020-0466-9 (PMC7714695; doi:10.1038/s41594-020-0466-9)
Supplement: Supplementary file 1 — Supplementary Fig. 1 and Note. [file 41594_2020_466_MOESM1_ESM.pdf]

nature structural & molecular biology

SUPPLEMENTARY INFORMATION

https://doi.org/10.1038/s41594-020-0466-9

In the format provided by the authors and unedited.

The nucleosome acidic patch and H2A ubiquitination underlie mSWI/SNF recruitment in synovial sarcoma

In the format provided by the authors and unedited

Figure 1A

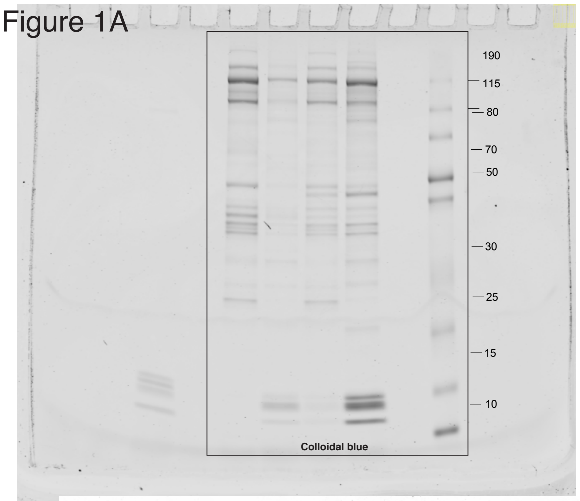

Figure 1C

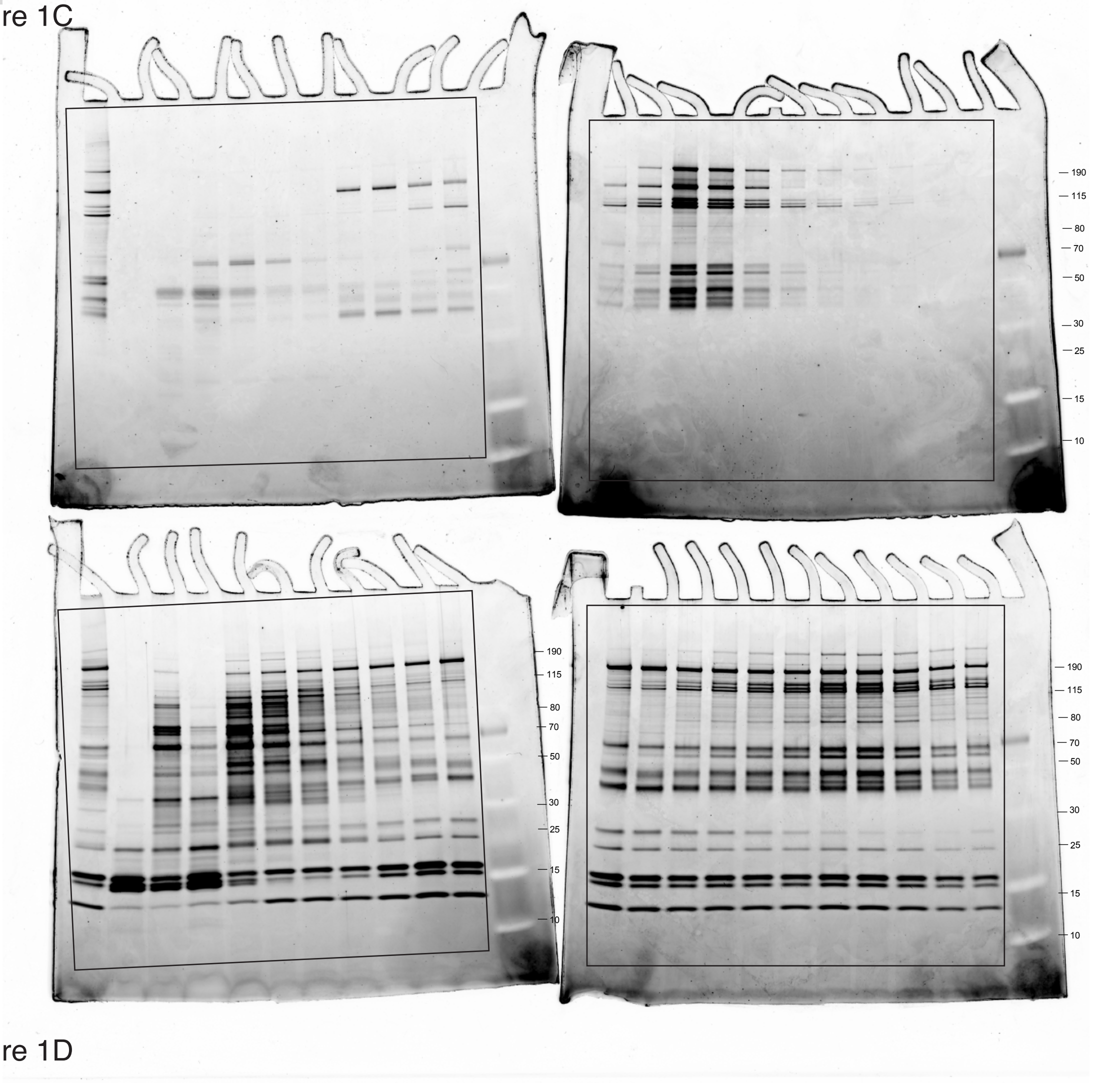

Figure 1D

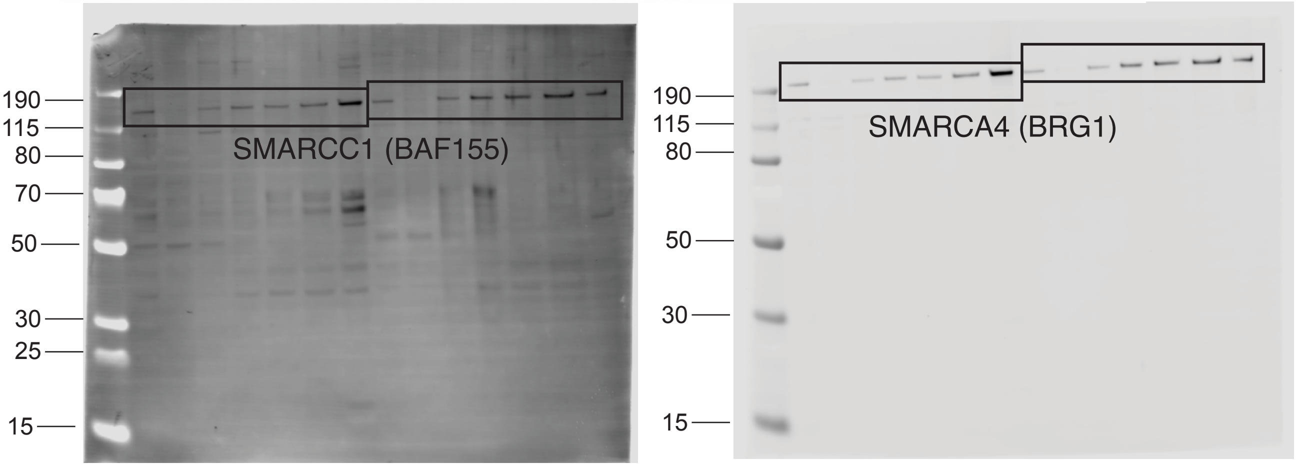

Extended Data Figure 1E

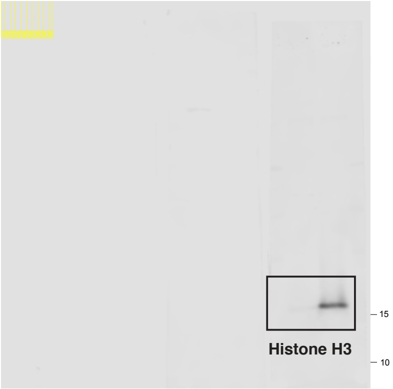

Extended Data Figure 1F

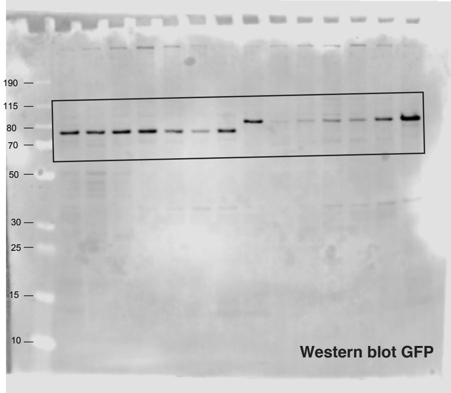

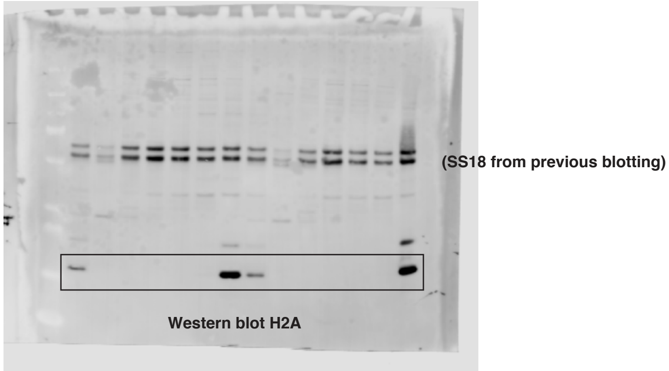

Extended Data Figure 1G

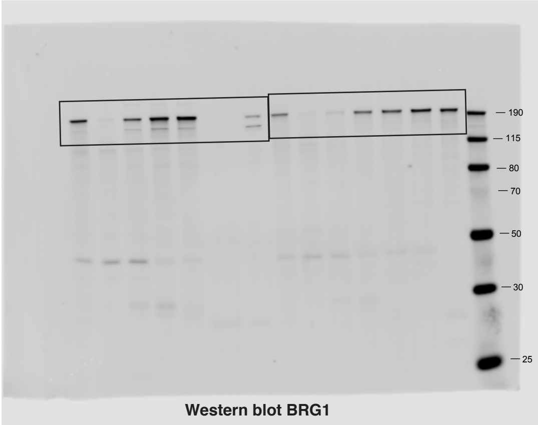

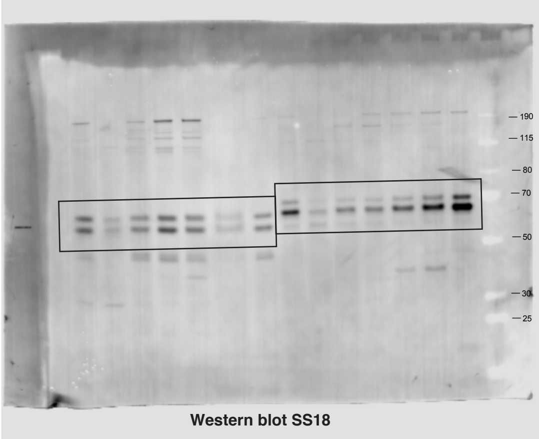

Figure 2A

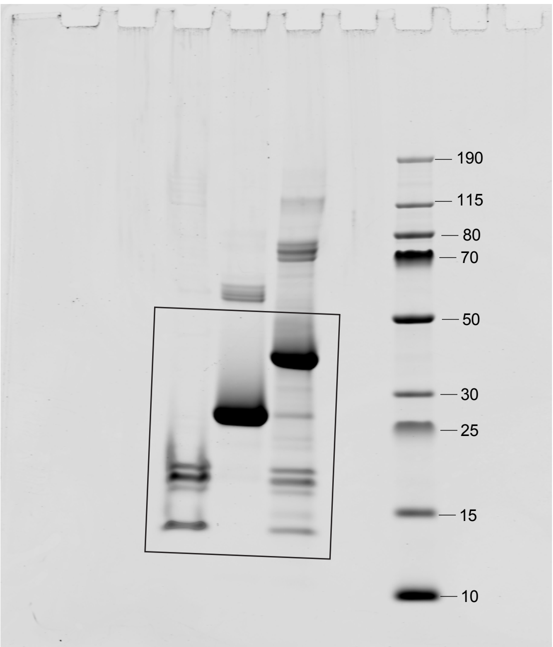

Colloidal blue

Figure 2E

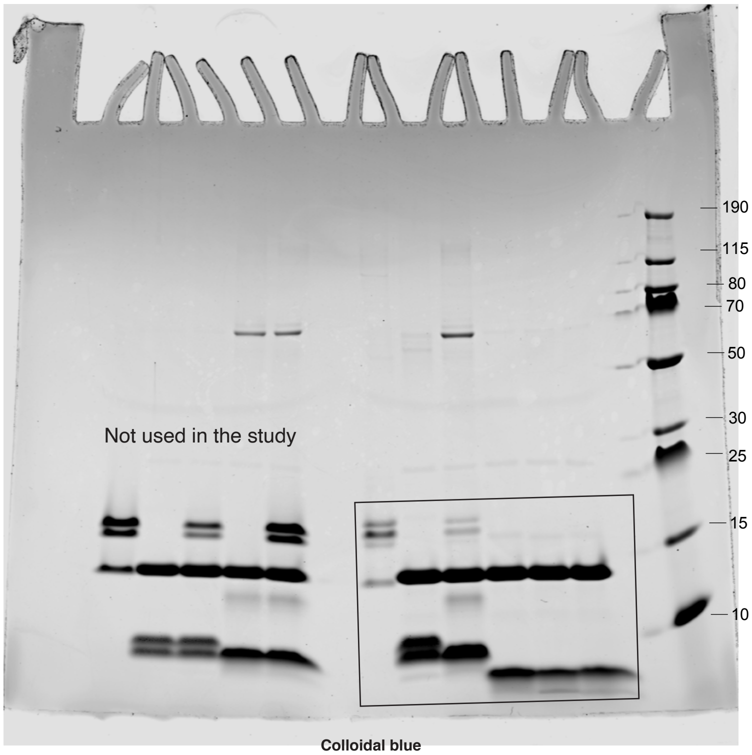

Colloidal blue

Figure 2F

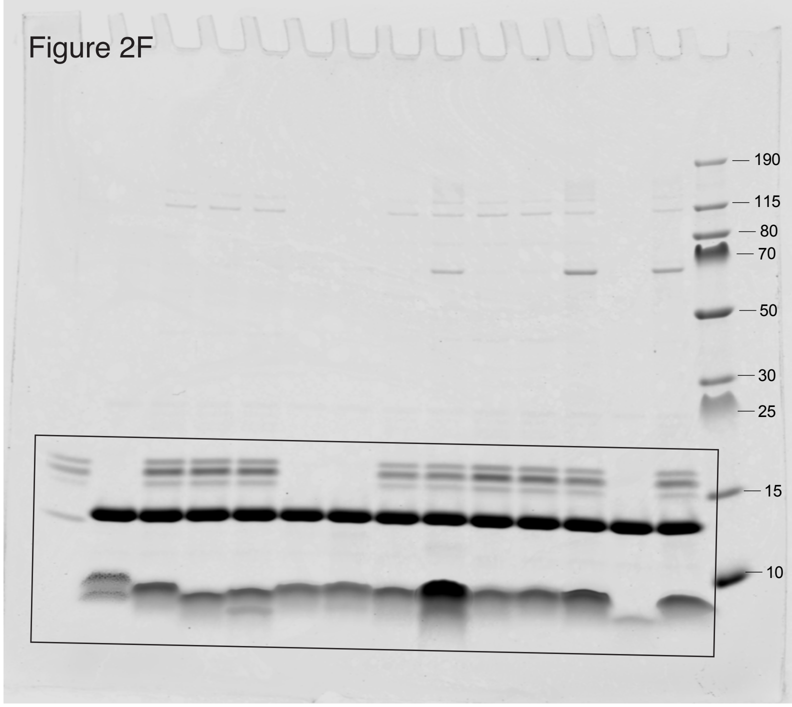

Colloidal blue

Extended Data Figure 2A

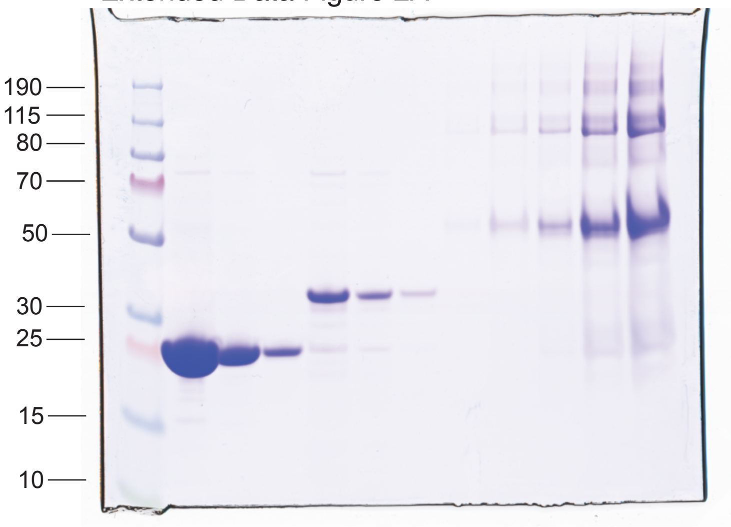

Extended Data Figure 2B

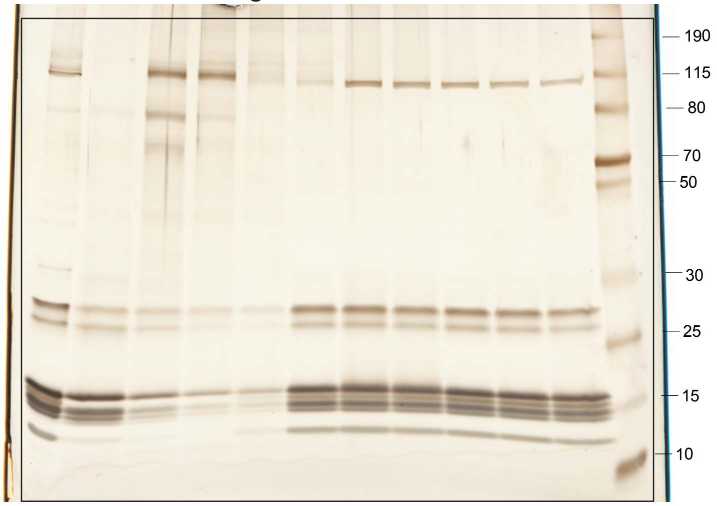

Silver stain

Upper panel

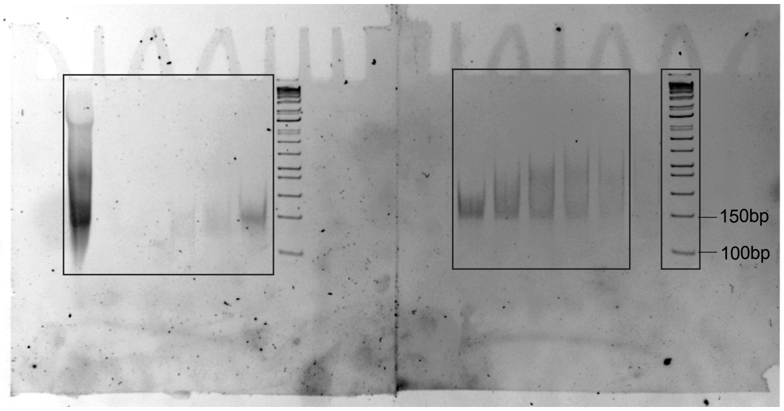

Sybr Gold

Lower panel

Extended Data 2C, top panel

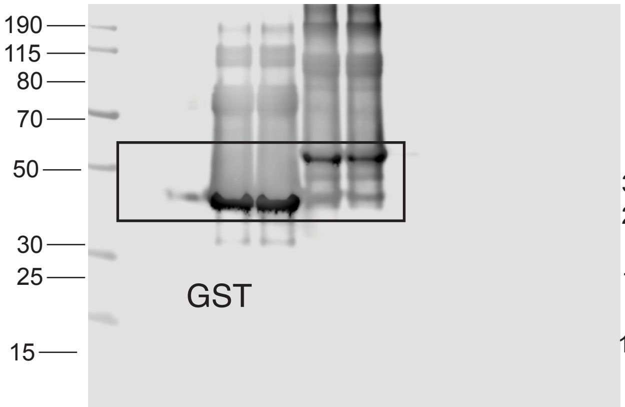

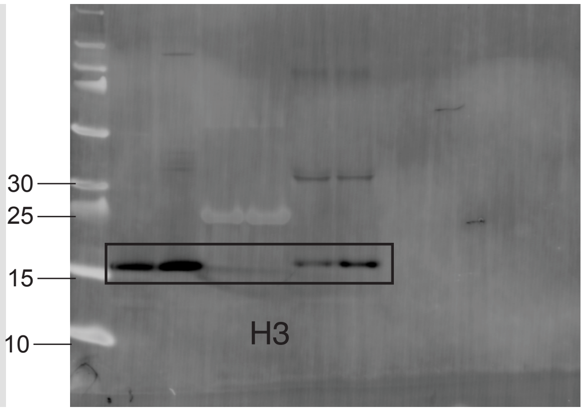

Extended Data 2C, bottom panel

Extended Data Figure 2E

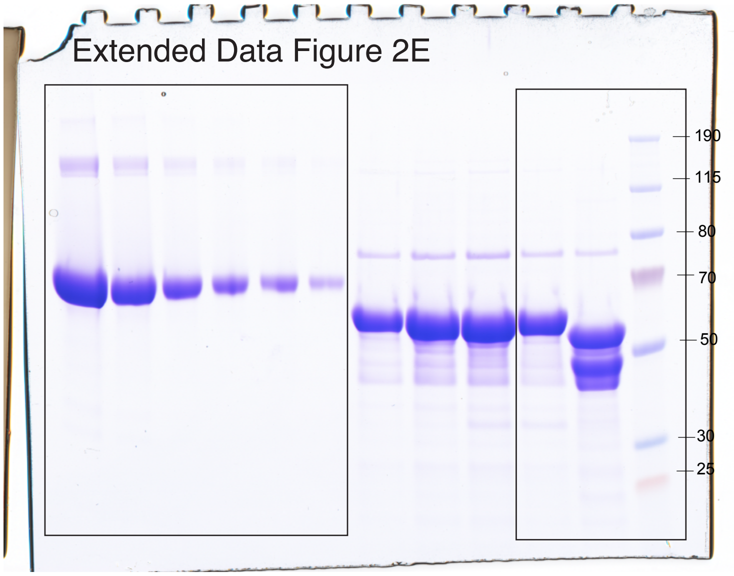

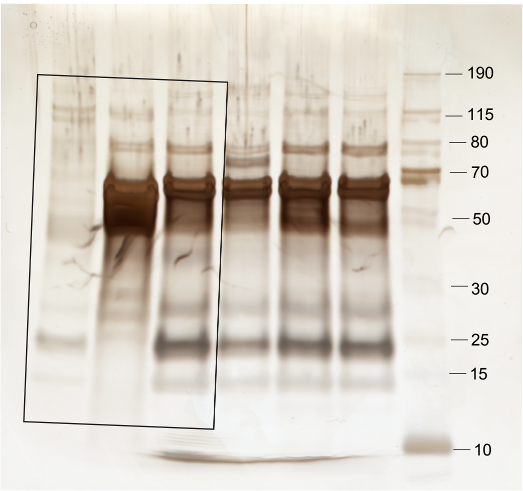

Coomassie

Figure 3A

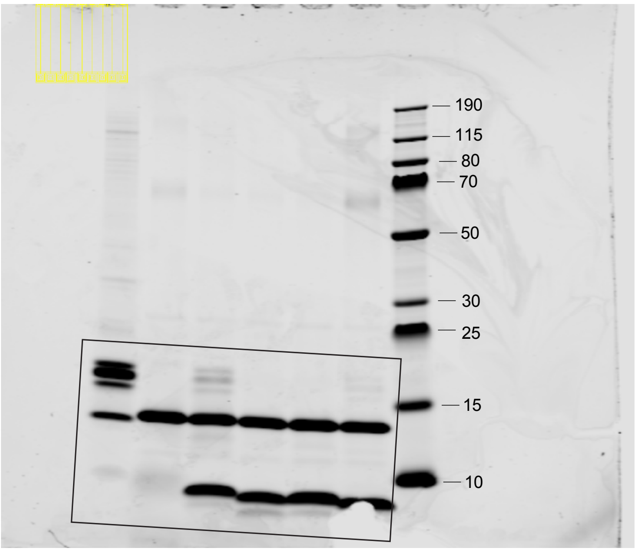

Colloidal blue

Figure 3B

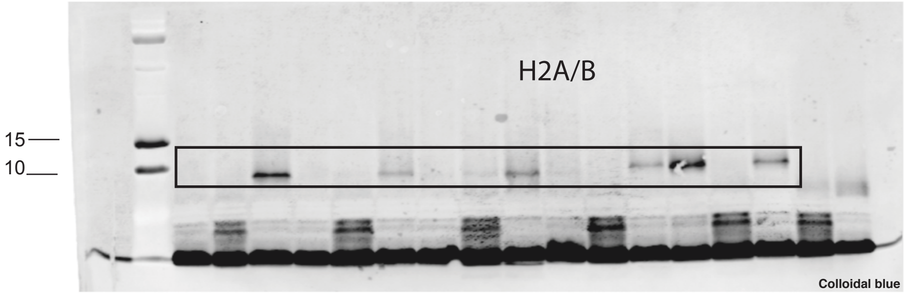

Figure 3D

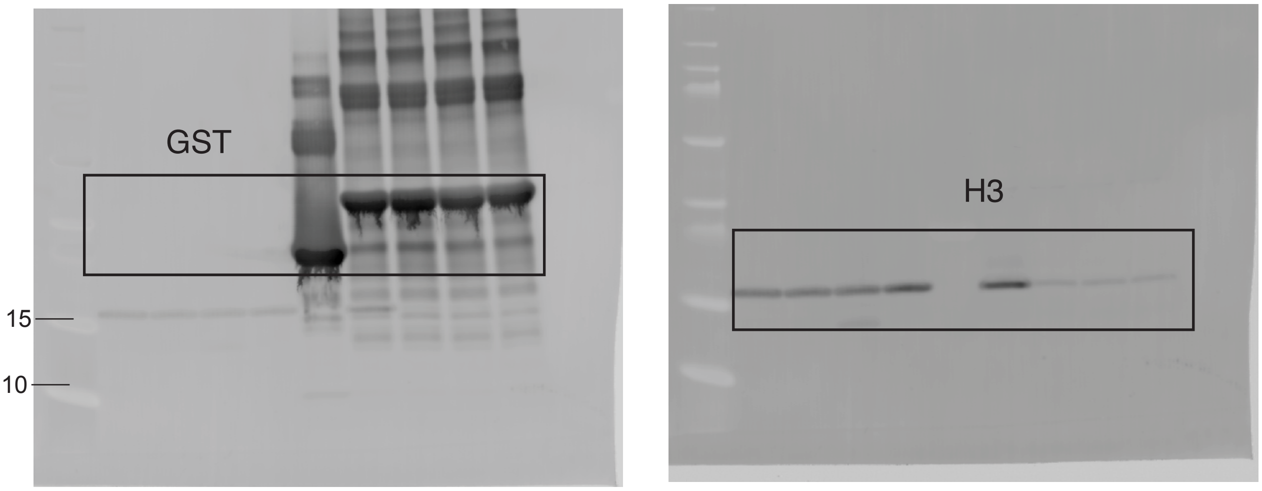

Figure 3E

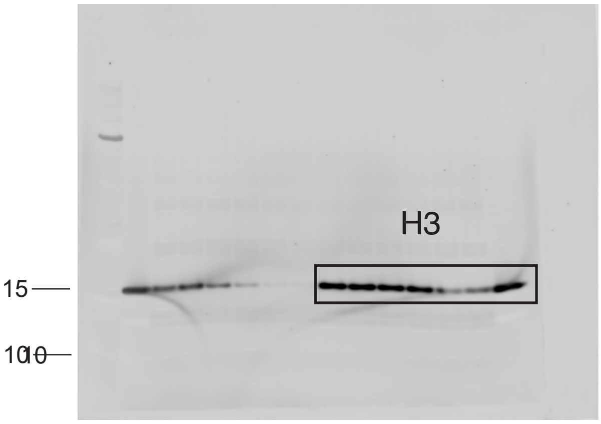

Figure 3H

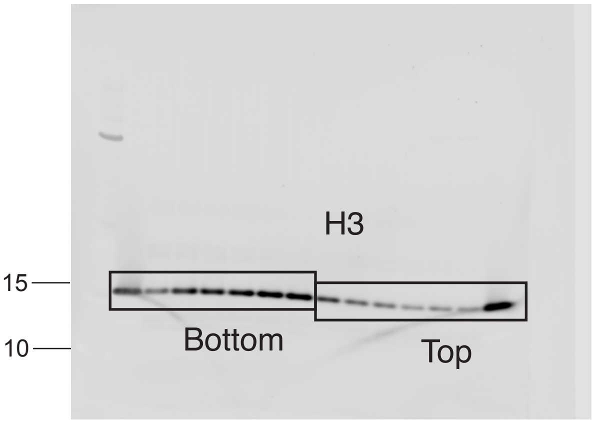

Extended Data Figure 3C

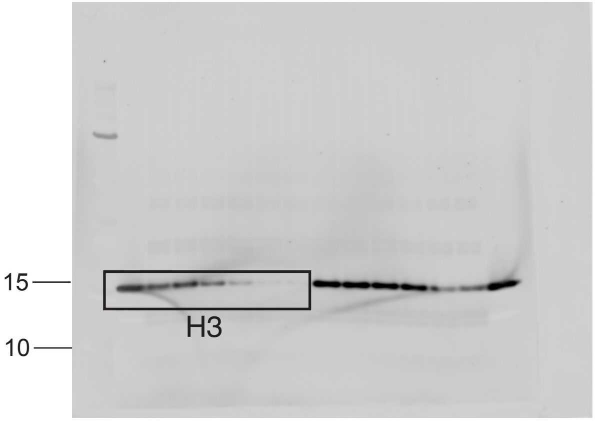

Extended Data Figure 3D

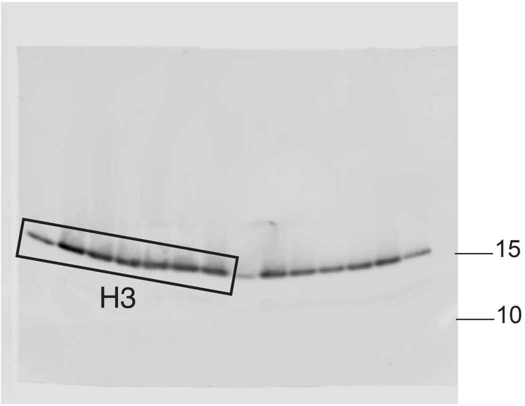

Extended Data Figure 3F

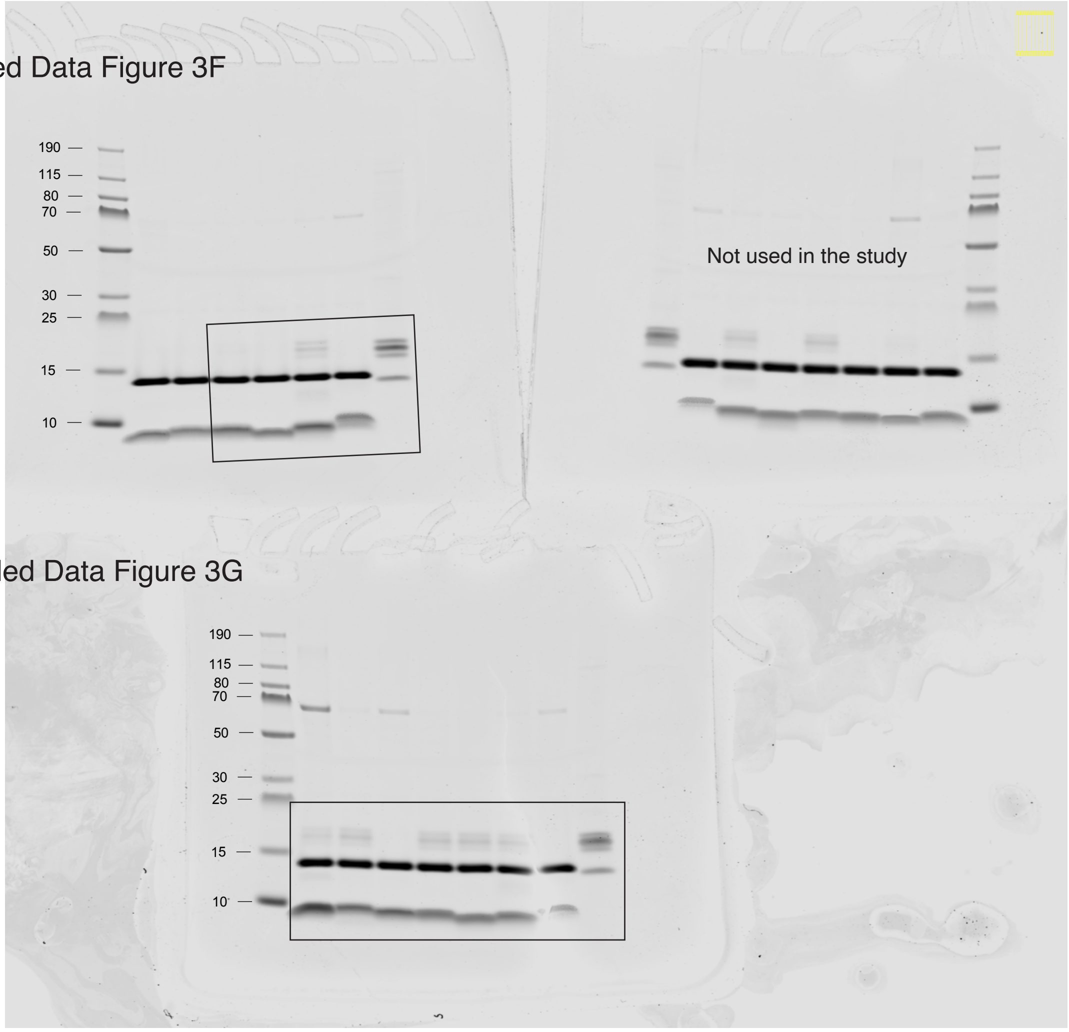

Colloidal blue

Extended Data Figure 4B (left)

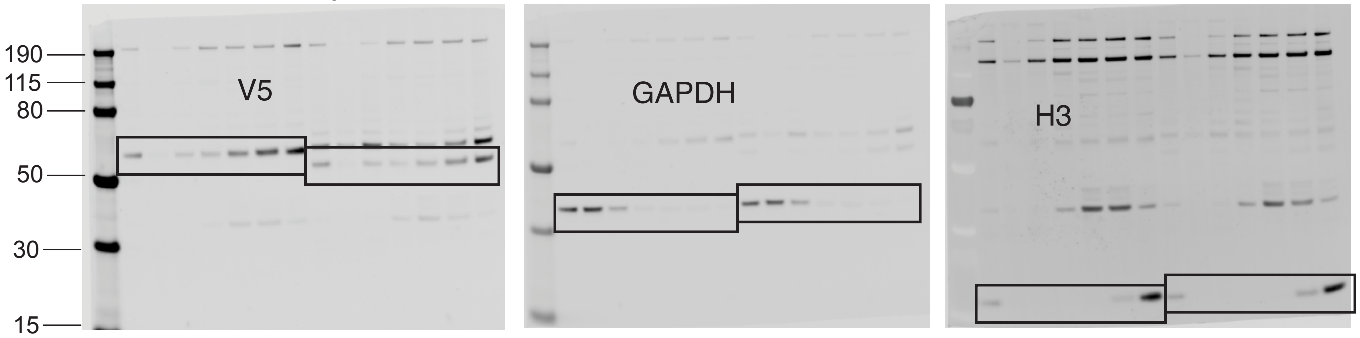

Extended Data Figure 4B (right)

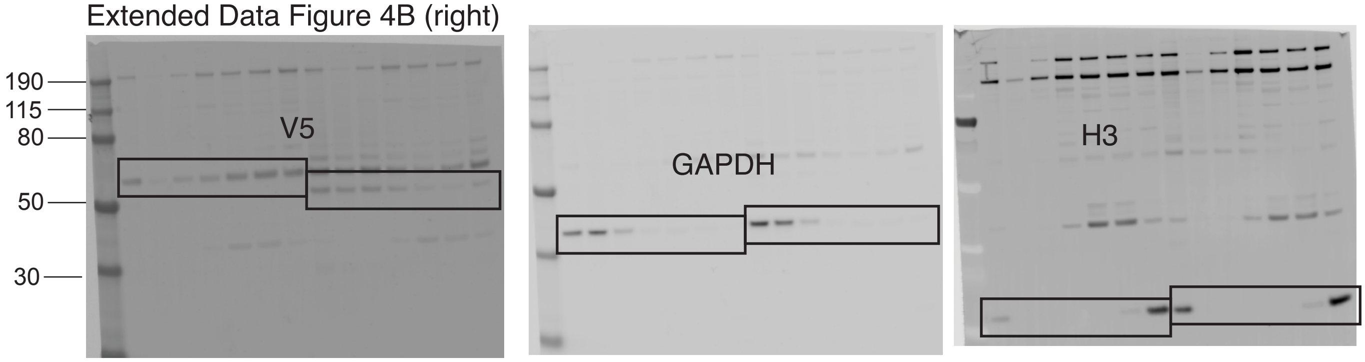

Extended Data Figure 4E [shSSX proliferation rescue blot]

Extended Data Figure 4E [shSSX proliferation rescue blots]

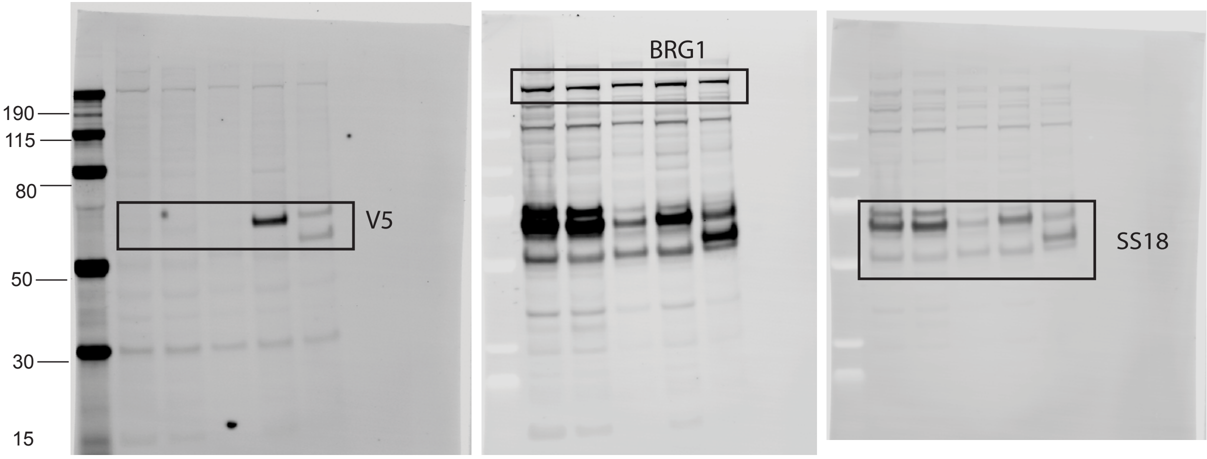

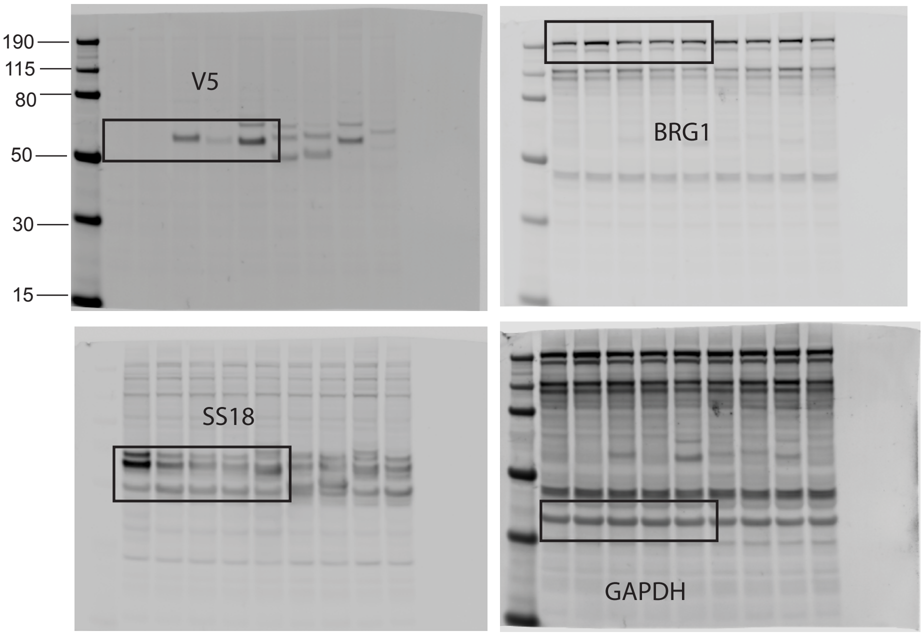

Extended Data Figure 4E [shSSX proliferation rescue blot, continued]

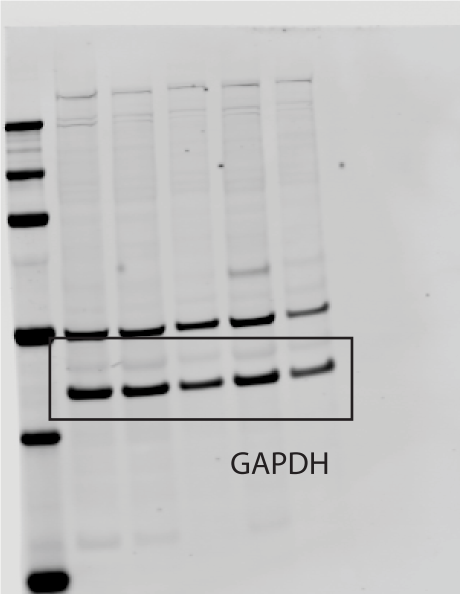

Figure 4D

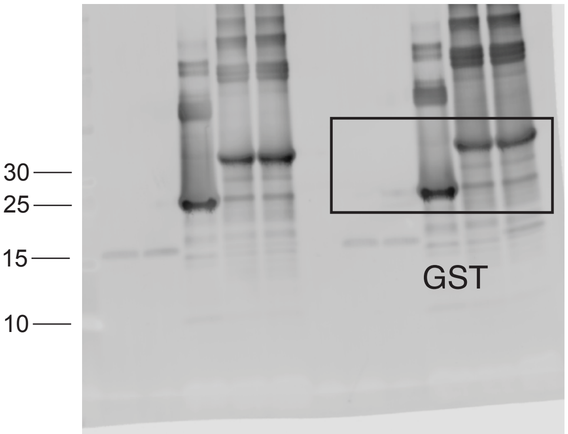

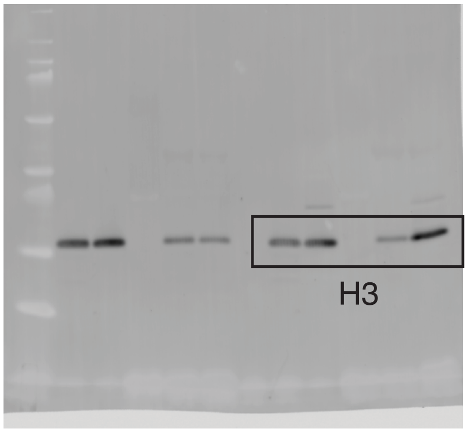

Figure 4F and Ext. Data Fig. 8G

Figure 4F (rep 1)

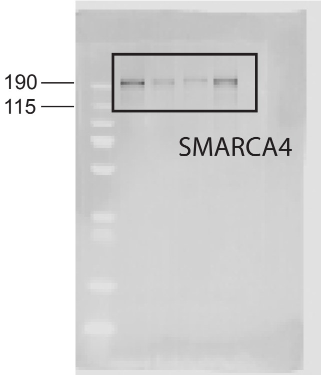

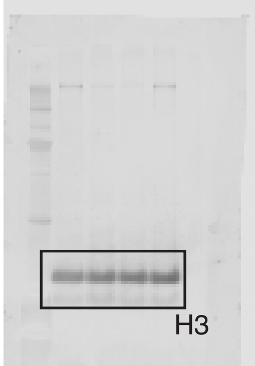

Blot 2 (rep 2)

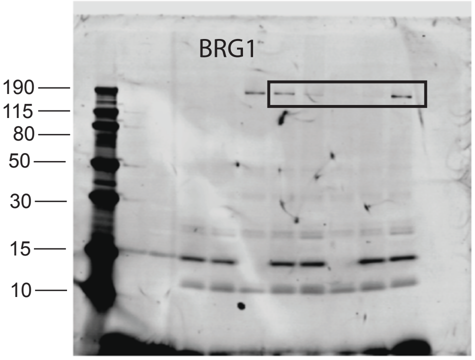

Ext. Data Fig. 8G

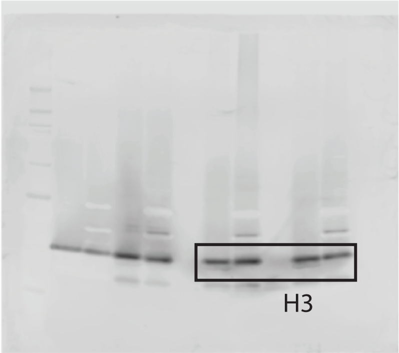

Blot 3 (rep 3)

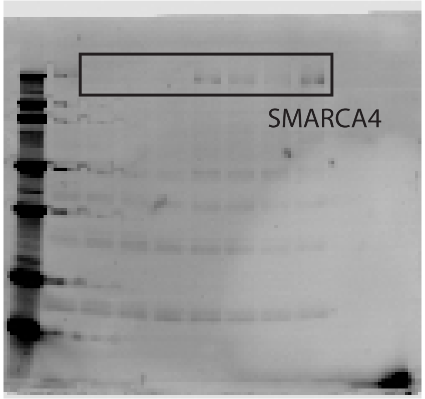

Ext. Data Fig. 8G

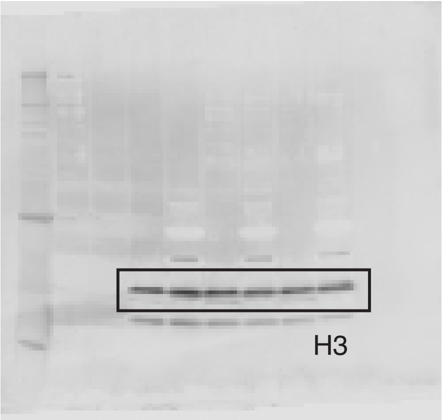

Blot 4 (rep 4)

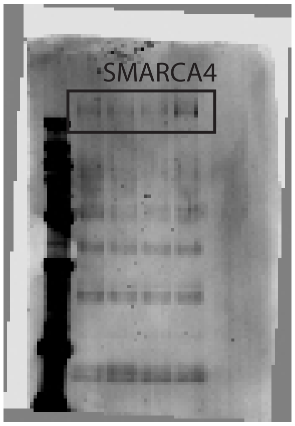

Ext. Data Fig. 8G

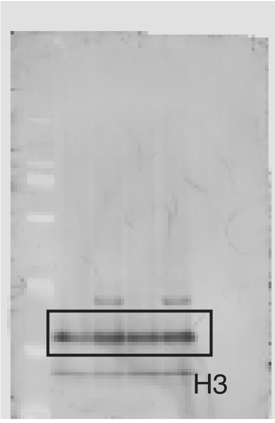

Blot 1 (rep 5) Ext. Data Fig. 8G

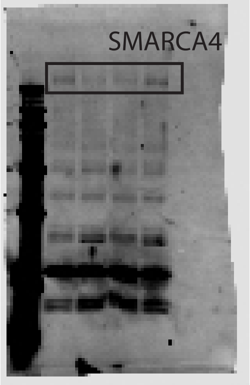

Figure 4G

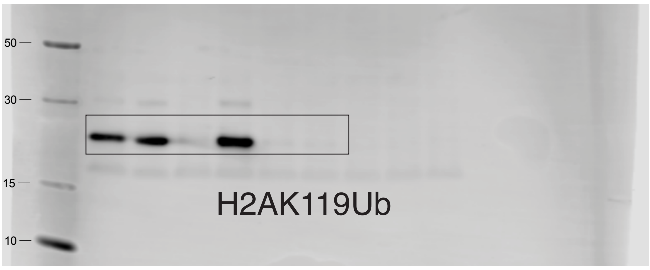

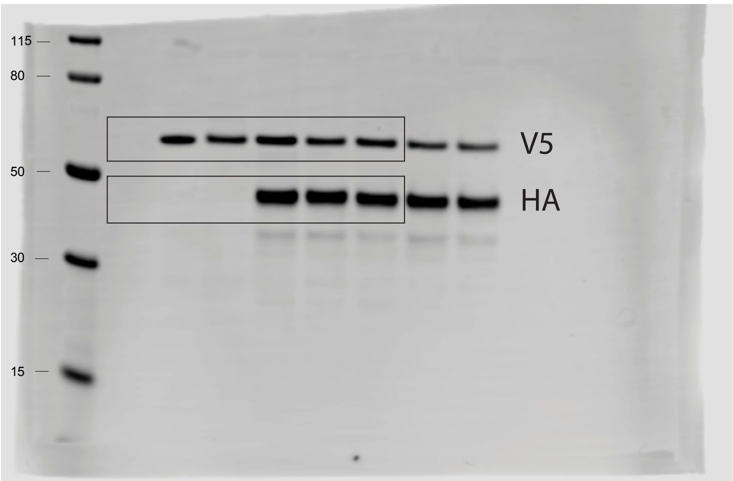

Figure 4J

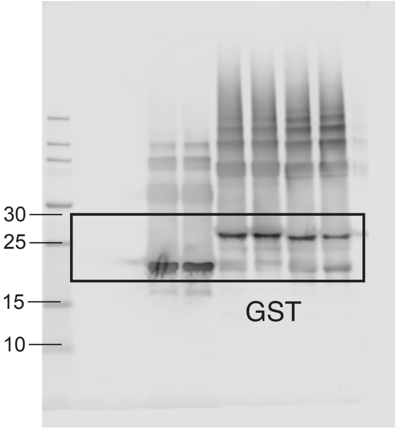

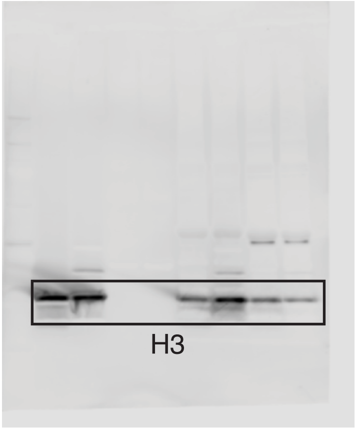

Extended Data Figure 5B

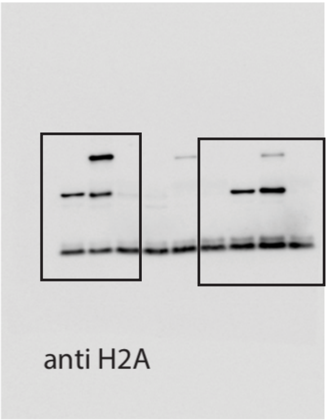

Extended Data Figure 5C

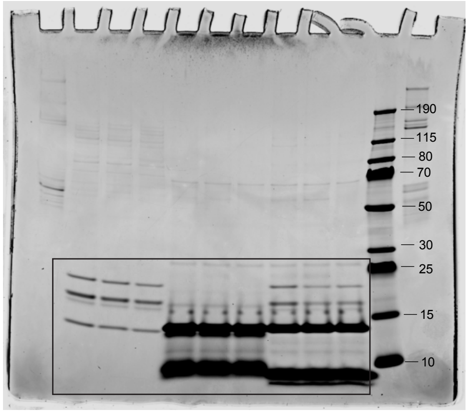

Colloidal blue

Extended Data Figure 6C

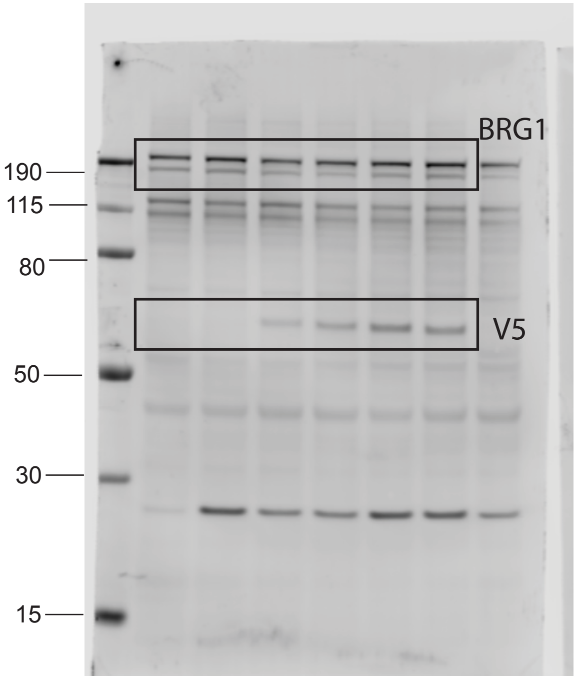

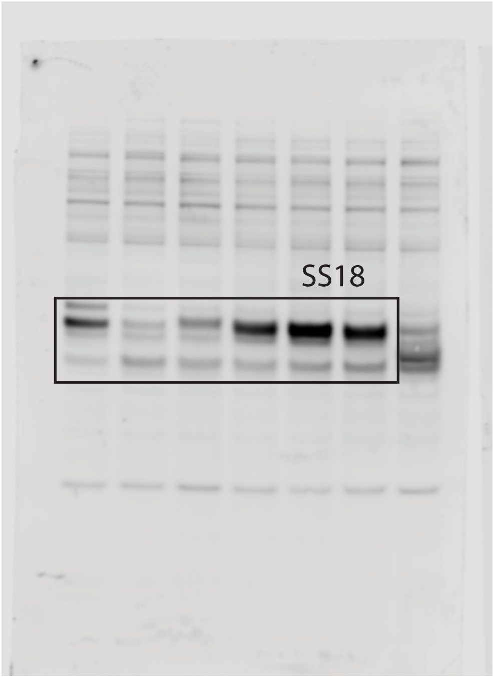

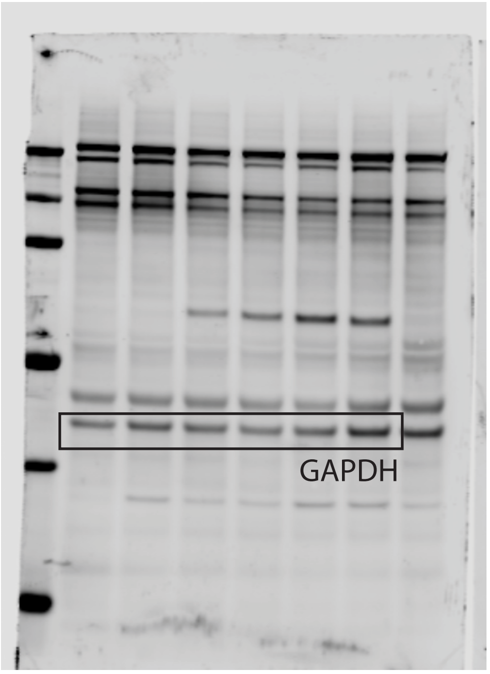

Extended Data Figure 7D [Left, V5-SS18-SSX mutant V5-IPs]

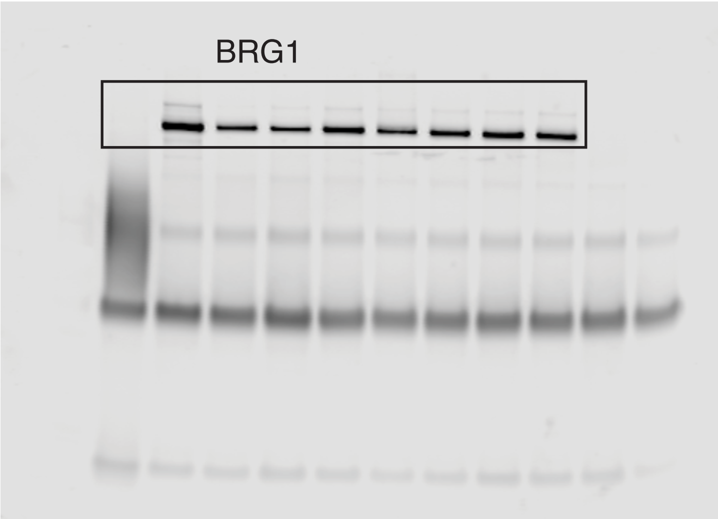

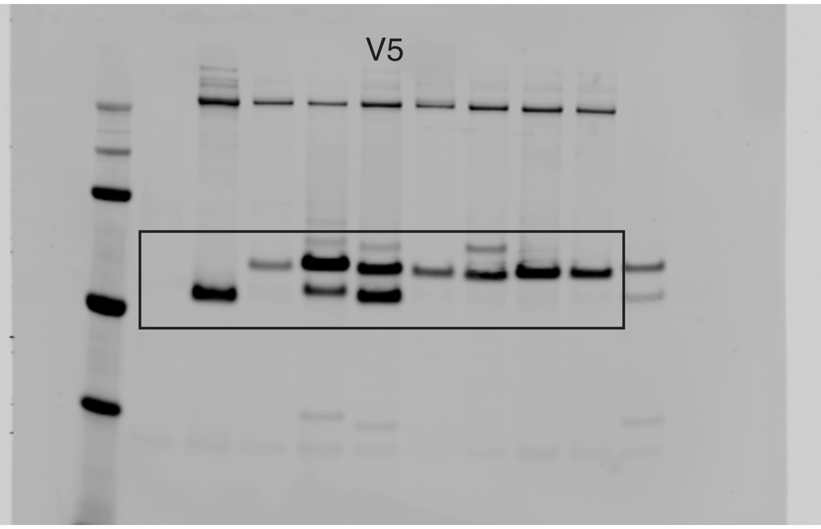

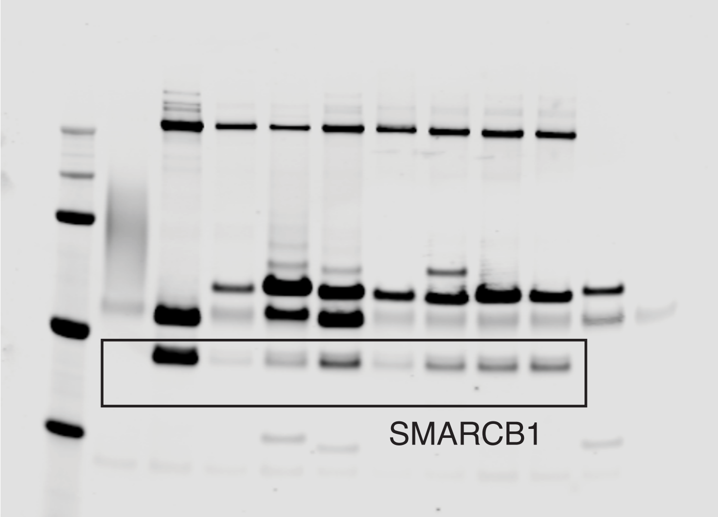

Extended Data Figure 7E

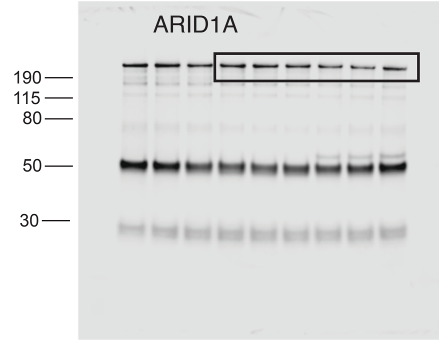

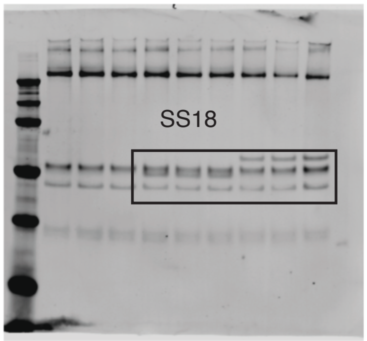

Extended Data Figure 7D [Right, V5-SS18-SSX mutant inputs]

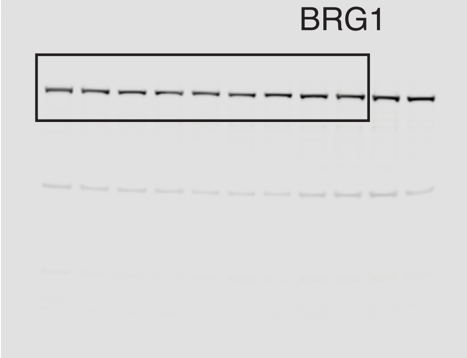

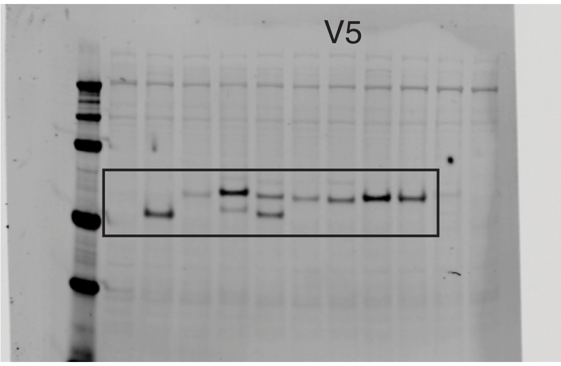

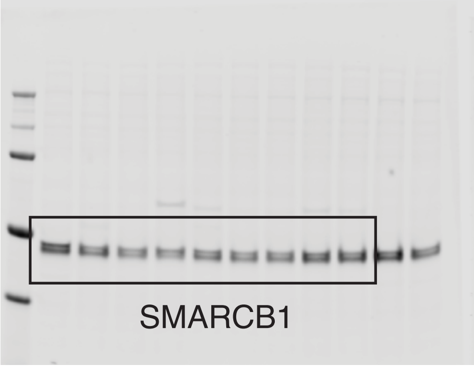

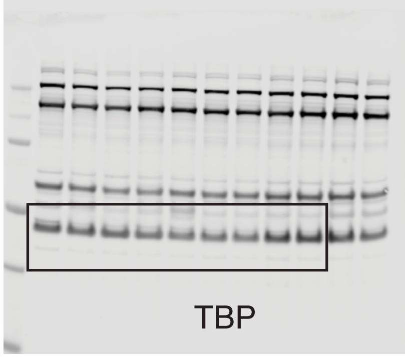

Extended Data Figure 8F

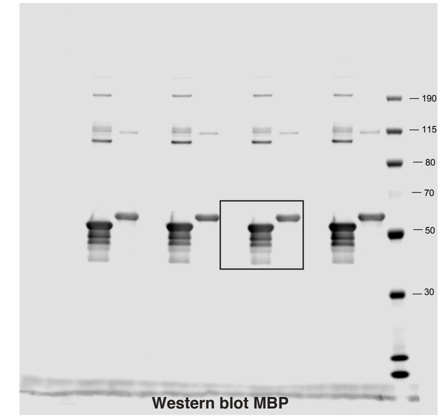

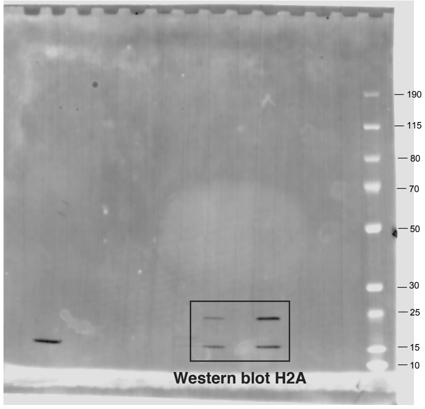

Extended Data Figure 8H

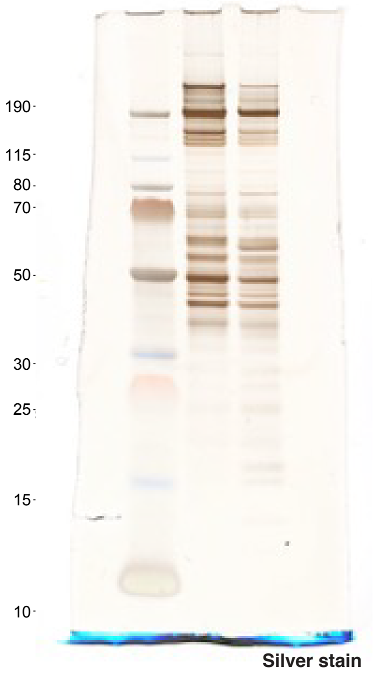

Extended Data Figure 9B

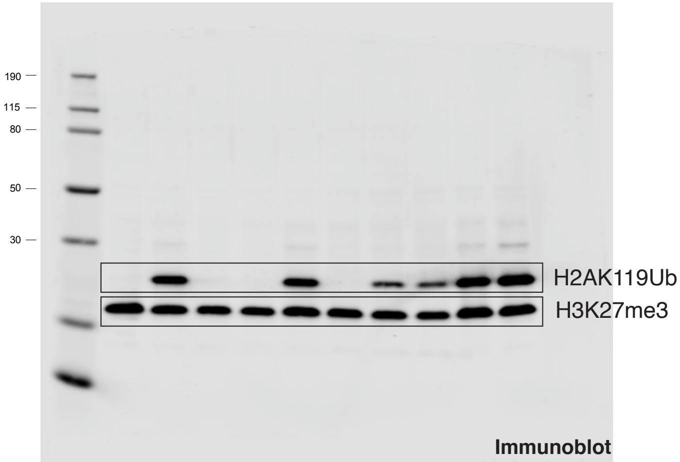

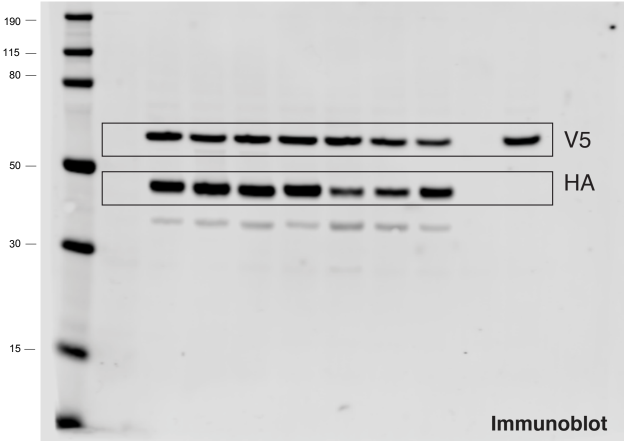

Extended Data Figure 9G

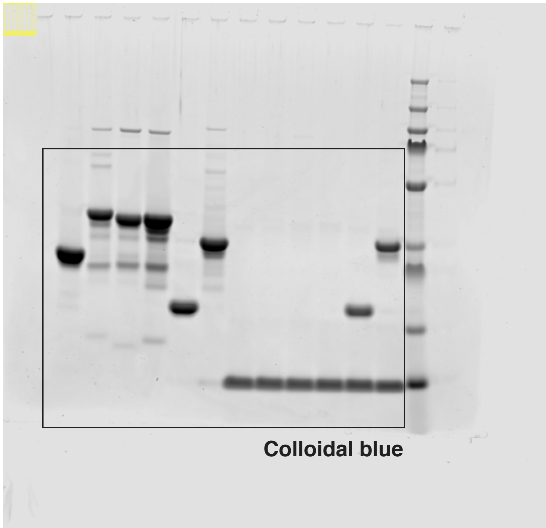

Supplementary Note
McBride, Mashtalir et al., 2020

Data Processing and Visualization for ChIP Samples

Alignment of ChIP-seq data was done using Bowtie2, version 2.1.0[1] and reads were mapped to the hg19 human reference genome, using the parameter –k 1.

To process the aligned data, peaks were called using MACS2[2] version 2.1.0 against an input sample with a $q = 0.001$ cutoff and broad peaks were called for each antibody in each cell line and condition. Those peaks that mapped to unmappable chromosomes (any that were not chr1–22, X or Y) or were located in blacklisted regions of ENCODE were excluded. For downstream analysis of data, bam files were generated with duplicates removed using the samtools rmdup command and the –b option. All ChIP-seq tracks were obtained from the bedGraphToBigWig script (UCSC) using bedgraph files generated with MACS2 using the –B –SPMR options. ChIP-seq tracks were visualized using IGV version 2.4.16 (Broad Institute).

To identify peaks of BAF complex localization, the merged peak set for V5 in V5-SS18 WT and V5-SS18-SSX1 conditions was used with bedtools merge –d 2000 to cause neighboring broad peaks to be called as a single peak. Read counts across peak sets were determined by calling the Rsubread v1.26.1 bioconductor package function featureCounts() on bam files. Subsequently, these values were divided by the total number of mapped reads divided by one million to give a normalized value of RPM for each interval contained within the input bed.

HTSeq was used to calculate metagene read densities with fragment lengths of 200bp to account for fragment size selection that occurs during sonication. Total read counts for each region was normalized by the number of mapped reads to calculate reads per million mapped reads. The metagene plots were created using mean read densities over all sites for each condition around the center of the peak. All ChIP-seq heatmaps were created using these same HTSeq read densities with sites were then ranked by mean ChIP-seq signal for the indicated antibody and condition. Heatmap visualization was obtained from Python matplotlib using a midpoint of 0.5 reads per million to set the threshold of visualization for the heatmap color scale.

Data Processing and Visualization for RNA Samples

STAR was used to determine RPM values for each sample. Significance was determined with the DESeq2 R package with input raw read counts obtained from Rsubread featureCounts against the hg19 refFlat annotation. Small RNA genes (MIR & SNO) were filtered out from the gene lists for all analyses. Genes with a significant change in expression were determined with a Bonferri-corrected p-value of less than 1e-5, a two-fold change in gene expression (|log2FC|>1), and inclusion of expressed genes (RPKM ≥ 1 in a minimum of one sample) to identify significantly changing genes. For visualization of RNA-seq data, heatmaps were generated by plotting the z-scores of RPKM values across each sample of the comparison conditions.

References:

1. Langmead, B. & Salzberg, S.L. Fast gapped-read alignment with Bowtie 2. *Nat Methods* **9**, 357-9 (2012).
2. Zhang, Y. et al. Model-based analysis of ChIP-Seq (MACS). *Genome Biol* **9**, R137 (2008).
