# Supplementary material for: The nucleosome acidic patch and H2A ubiquitination underlie mSWI/SNF recruitment in synovial sarcoma
Source: Nat Struct Mol Biol. 2020 Aug 3;27(9):836–45. doi: 10.1038/s41594-020-0466-9 (PMC7714695; doi:10.1038/s41594-020-0466-9)
Supplement: Supplementary file 2 — Reporting Summary [file 41594_2020_466_MOESM2_ESM.pdf]

nature research

Corresponding author(s): Cigall Kadoch, Ph.D.

Last updated by author(s): 03-31-2020

Reporting Summary

Nature Research wishes to improve the reproducibility of the work that we publish. This form provides structure for consistency and transparency in reporting. For further information on Nature Research policies, see Authors & Referees and the Editorial Policy Checklist.

Statistics

For all statistical analyses, confirm that the following items are present in the figure legend, table legend, main text, or Methods section.

n/a	Confirmed	
☐	☒	The exact sample size (n) for each experimental group/condition, given as a discrete number and unit of measurement
☐	☒	A statement on whether measurements were taken from distinct samples or whether the same sample was measured repeatedly
☐	☒	The statistical test(s) used AND whether they are one- or two-sided<br>*Only common tests should be described solely by name; describe more complex techniques in the Methods section.*
☒	☐	A description of all covariates tested
☒	☐	A description of any assumptions or corrections, such as tests of normality and adjustment for multiple comparisons
☐	☒	A full description of the statistical parameters including central tendency (e.g. means) or other basic estimates (e.g. regression coefficient) AND variation (e.g. standard deviation) or associated estimates of uncertainty (e.g. confidence intervals)
☒	☐	For null hypothesis testing, the test statistic (e.g. F, t, r) with confidence intervals, effect sizes, degrees of freedom and P value noted<br>*Give P values as exact values whenever suitable.*
☒	☐	For Bayesian analysis, information on the choice of priors and Markov chain Monte Carlo settings
☒	☐	For hierarchical and complex designs, identification of the appropriate level for tests and full reporting of outcomes
☒	☐	Estimates of effect sizes (e.g. Cohen's d, Pearson's r), indicating how they were calculated

Our web collection on statistics for biologists contains articles on many of the points above.

Software and code

Policy information about availability of computer code

Data collection	All packages used for data analysis and collection are listed below in ChIP-Seq section.
Data analysis	All analysis packages used for data analysis and collection are detailed below in the Methods section (under ChIP-Seq, RNA-seq, ATAC-seq analyses); all code is deposited on GitHub at https://github.com/ardavino/

For manuscripts utilizing custom algorithms or software that are central to the research but not yet described in published literature, software must be made available to editors/reviewers. We strongly encourage code deposition in a community repository (e.g. GitHub). See the Nature Research guidelines for submitting code & software for further information.

Data

Policy information about availability of data

All manuscripts must include a data availability statement. This statement should provide the following information, where applicable:

- Accession codes, unique identifiers, or web links for publicly available datasets
- A list of figures that have associated raw data
- A description of any restrictions on data availability

All data is deposited under Gene Expression Omnibus GSE139055, to be made accessible on the day of publication.

Field-specific reporting

Please select the one below that is the best fit for your research. If you are not sure, read the appropriate sections before making your selection.

☒ Life sciences ☐ Behavioural & social sciences ☐ Ecological, evolutionary & environmental sciences

Life sciences study design

All studies must disclose on these points even when the disclosure is negative.

Sample size	No sample-size calculations were performed; all experiments are performed in technical and experimental (of at least n>=3 replicates) across the cell lines most appropriate for the evaluation of the biologic inquiries at hand.
Data exclusions	No data exclusions were executed in this study.
Replication	Extensive measures were taken to verify reproducibility of results, including the utility of mutliple cell lines, where appropriate, multiple experiments performed by different individuals, and consistent validation of constructs used. All data presented in this manuscript have been replicated in > n=3 independent experiments.
Randomization	No randomization was performed in this study. N/A
Blinding	N/A, no blinding to group allocation.

Reporting for specific materials, systems and methods

We require information from authors about some types of materials, experimental systems and methods used in many studies. Here, indicate whether each material, system or method listed is relevant to your study. If you are not sure if a list item applies to your research, read the appropriate section before selecting a response.

Materials & experimental systems

n/a	Involved in the study	
☐	☒	Antibodies
☐	☒	Eukaryotic cell lines
☒	☐	Palaeontology
☒	☐	Animals and other organisms
☒	☐	Human research participants
☒	☐	Clinical data

Methods

n/a	Involved in the study	
☐	☒	ChIP-seq
☒	☐	Flow cytometry
☒	☐	MRI-based neuroimaging

Antibodies

Antibodies used	Anti-SMARCA4 (Cell Signaling Technology), Anti-SMARCC1 (Cell Signaling Technology), Anti-SS18 (Cell Signaling Technology), Anti-GFP (Invitrogen), anti-V5 (Cell Signaling Technology), anti-GAPDH (Santa Cruz Biotechnology), anti-Histone H3 (Abcam), Anti-ARID1A (Cell Signaling Technology), anti-H2A Ub119 (Cell Signaling Technology), anti-H3K27me3 (Abcam), anti-RING1B (Cell Signaling Technology), anti-BAF47 (Santa Cruz Biotechnology), anti-GST (Sigma-Aldrich), anti-HA (Abcam), and anti-MBP (New England Biolabs).
Validation	All primary antibodies used in this study were validated in-house for on-target specificity using cell lines in which the target was knocked out or genetically deleted. These studies were performed in addition to the manufacturer's specifications.

Eukaryotic cell lines

Policy information about cell lines

Cell line source(s)	All cell lines used are available from ATCC or RIKEN repositories.
Authentication	All cell lines were subjected to standard cell line fingerprinting analyses via our in-house cell line identify verification pipeline (Dana-Farber Molecular Diagnostics Laboratory).
Mycoplasma contamination	All cell lines used in this study were routinely tested for mycoplasma contamination and were confirmed to be negative throughout the study.
Commonly misidentified lines (See ICLAC register)	N/A

ChIP-seq

Data deposition

☒ Confirm that both raw and final processed data have been deposited in a public database such as GEO.

☒ Confirm that you have deposited or provided access to graph files (e.g. BED files) for the called peaks.

Data access links<br>*May remain private before publication.*	All data is deposited under Gene Expression Omnibus GSE139055<br>https://www.ncbi.nlm.nih.gov/geo/query/acc.cgi?acc=GSE139055<br>Reviewer token: stwpuioirhkvzsb
Files in database submission	GSM4128405 CRL7250_V5-SS18-SSX_7aa_AcidicDel_V5_ChIP-Seq<br>GSM4128406 CRL7250_V5-SS18-SSX_6aa_BasicDel_V5_ChIP-Seq<br>GSM4128407 CRL7250_V5-SS18-SSX_23aa-SSX-Tail_V5_ChIP-Seq<br>GSM4128408 CRL7250_V5-SS18-SSX_33aa-SSX-Tail_V5_ChIP-Seq<br>GSM4128417 CLR7250_V5-SS18-SSX_78aa-SSX-Tail_Input_ChIP-Seq<br>GSM4128418 CLR7250_V5-SS18-SSX_78aa-SSX-Tail_V5_ChIP-Seq<br>GSM4128419 CLR7250_V5-SS18-SSX_W164A-mut-SSX-Tail_Input_ChIP-Seq<br>GSM4128420 CLR7250_V5-SS18-SSX_W164A-mut-SSX-Tail_V5_ChIP-Seq<br>GSM4128421 CLR7250_V5-SS18-SSX_L168A-mut-SSX-Tail_Input_ChIP-Seq<br>GSM4128422 CLR7250_V5-SS18-SSX_L168A-mut-SSX-Tail_V5_ChIP-Seq<br>GSM4128423 CLR7250_V5-SS18-SSX_33aa-SSX-Tail_repeat_Input_ChIP-Seq<br>GSM4128424 CLR7250_V5-SS18-SSX_33aa-SSX-Tail_repeat_V5_ChIP-Seq<br>GSM4128425 SYO1_shCt_Input_ChIP-Seq<br>GSM4128427 SYO1_shCt_SS18_ChIP-Seq<br>GSM4128433 SYO1_shCt_RING1B_ChIP-Seq<br>GSM4128437 SYO1_shCt_H2AUb1K119_ChIP-Seq<br>GSM4128450 SYO1_shCt_H2AK119Ub_ChIP-Seq
Genome browser session<br>(e.g. UCSC)	N/A

Methodology

Replicates	All experiments were performed in at least two experimental replicates
Sequencing depth	Sample Mapped Reads Total Reads<br>CRL7250_V5-SS18-SSX_7aa_AcidicDel_V5_ChIP-Seq 15297180 18491697<br>CRL7250_V5-SS18-SSX_6aa_BasicDel_V5_ChIP-Seq 26959409 28741367<br>CRL7250_V5-SS18-SSX_23aa-SSX-Tail_V5_ChIP-Seq 22132127 23720917<br>CRL7250_V5-SS18-SSX_33aa-SSX-Tail_V5_ChIP-Seq 14292546 14800795<br>CLR7250_V5-SS18-SSX_78aa-SSX-Tail_Input_ChIP-Seq 36922119 37796567<br>CLR7250_V5-SS18-SSX_78aa-SSX-Tail_V5_ChIP-Seq 21126052 24887406<br>CLR7250_V5-SS18-SSX_W164A-mut-SSX-Tail_Input_ChIP-Seq 39042088 40027808<br>CLR7250_V5-SS18-SSX_W164A-mut-SSX-Tail_V5_ChIP-Seq 20246691 26407440<br>CLR7250_V5-SS18-SSX_L168A-mut-SSX-Tail_Input_ChIP-Seq 51623232 52983471<br>CLR7250_V5-SS18-SSX_L168A-mut-SSX-Tail_V5_ChIP-Seq 21295373 28731528<br>CLR7250_V5-SS18-SSX_33aa-SSX-Tail_repeat_Input_ChIP-Seq 43059605 44214889<br>CLR7250_V5-SS18-SSX_33aa-SSX-Tail_repeat_V5_ChIP-Seq 30484351 34812551<br>SYO1_shCt_Input_ChIP-Seq 16835065 17427910<br>SYO1_shCt_SS18_ChIP-Seq 25330796 26754873<br>SYO1_shCt_RING1B_ChIP-Seq 14835584 16895758<br>SYO1_shCt_H2AUb1 K119_ChIP-Seq 8388190 8997519<br>CRL7250_WT_Input_ChIP-Seq 25001725 25911100<br>CRL7250_WT_RING1B_ChIP-Seq 16363707 18048718<br>SYO1_shCt_H2AK119Ub_ChIP-Seq 19943172 20212804
Antibodies	CRL7250_V5-SS18-SSX_7aa_AcidicDel_V5_ChIP-Seq CST (D3H8Q)<br>CRL7250_V5-SS18-SSX_6aa_BasicDel_V5_ChIP-Seq CST (D3H8Q)<br>CRL7250_V5-SS18-SSX_23aa-SSX-Tail_V5_ChIP-Seq CST (D3H8Q)<br>CRL7250_V5-SS18-SSX_33aa-SSX-Tail_V5_ChIP-Seq CST (D3H8Q)<br>CLR7250_V5-SS18-SSX_78aa-SSX-Tail_Input_ChIP-Seq N/A<br>CLR7250_V5-SS18-SSX_78aa-SSX-Tail_V5_ChIP-Seq CST (D3H8Q)<br>CLR7250_V5-SS18-SSX_W164A-mut-SSX-Tail_Input_ChIP-Seq N/A<br>CLR7250_V5-SS18-SSX_W164A-mut-SSX-Tail_V5_ChIP-Seq CST (D3H8Q)<br>CLR7250_V5-SS18-SSX_L168A-mut-SSX-Tail_Input_ChIP-Seq N/A<br>CLR7250_V5-SS18-SSX_L168A-mut-SSX-Tail_V5_ChIP-Seq CST (D3H8Q)<br>CLR7250_V5-SS18-SSX_33aa-SSX-Tail_repeat_Input_ChIP-Seq N/A<br>CLR7250_V5-SS18-SSX_33aa-SSX-Tail_repeat_V5_ChIP-Seq CST (D3H8Q)<br>SYO1_shCt_Input_ChIP-Seq N/A<br>SYO1_shCt_SS18_ChIP-Seq CST (D6I4Z)

SYO1_shCt_RING1B_ChIP-Seq CST (D22F2)
SYO1_shCt_H2AUb1K119_ChIP-Seq CST (D27C4)
CRL7250_WT_Input_ChIP-Seq N/A
CRL7250_WT_RING1B_ChIP-Seq CST (D22F2)
SYO1_shCt_H2AK119Ub_ChIP-Seq CST (D27C4)

Peak calling parameters

Broad peaks were called for all BAF peaks and histone marks using MACS2 'callpeak' with a -q value of 1e-3, and the options --broad_cutoff --broad --nomodel --SPMR against an input sample.

Data quality

BAF and V5 peaks that were used for reference throughout the study had well over 15,000+ broad peaks in each sample with a stringent q value cut off of 1e-3.

Software

ChIP-Seq Data was aligned using Bowtie2, version 2.1.0 to the hg19 reference genome with the parameter k -1. Duplicates were removed from reads files using samtools rmdup with the -b option. (SAMtools v1.3.1). Heatmaps and metagene plots were generated created using HTSeq v0.9.1. Read count across peak sets of interest were calculated by calling the Rsubread v1.26.1 bioconductor package function featureCounts() on duplicate removed bam files.
